# Tumor suppressive BTB/POZ zinc-finger protein ZBTB28 inhibits oncogenic BCL6/ZBTB27 signaling to maintain p53 transcription in multiple carcinogenesis

**DOI:** 10.7150/thno.34983

**Published:** 2019-10-18

**Authors:** Tingxiu Xiang, Jun Tang, Lili Li, Weiyan Peng, Zhenfang Du, Xiangyu Wang, Qianqian Li, Hongying Xu, Lei Xiong, Can Xu, Xin Le, Xufu Wei, Fang Yu, Shuman Li, Qian Xiao, Bing Luo, Xinni Xiang, Ailong Huang, Yong Lin, Guosheng Ren, Qian Tao

**Affiliations:** 1 Key Laboratory of Molecular Oncology and Epigenetics, the First Affiliated Hospital of Chongqing Medical University, Chongqing, China;; 2 Cancer Epigenetics Laboratory, Department of Clinical Oncology, State Key Laboratory of Translational Oncology, Sir YK Pao Center for Cancer and Li Ka Shing Institute of Health Sciences, The Chinese University of Hong Kong, Hong Kong;; 3 Department of Medical Microbiology, Qingdao University Medical College, China;; 4 MOE Key Laboratory of Molecular Biology for Infectious Diseases, Department of Infectious Disease, Chongqing Medical University, China;; 5 Lovelace Respiratory Research Institute, Albuquerque, New Mexico, USA

**Keywords:** ZBTB28, CpG methylation, BCL6, p53, zinc finger protein

## Abstract

Zinc-finger and BTB/POZ domain-containing family proteins (ZBTB) are important transcription factors functioning as tumor suppressors or oncogenes, such as BCL6/ZBTB27 as a key oncoprotein for anti-cancer therapy. Through epigenome study, we identified ZBTB28/BCL6B/BAZF, a BTB/POZ domain protein highly homologous to BCL6, as a methylated target in multiple tumors. However, the functions and mechanism of ZBTB28 in carcinogenesis remain unclear. **Methods**: ZBTB28 expression and methylation were examined by reverse-transcription PCR and methylation-specific PCR. The effects and mechanisms of ectopic ZBTB28 expression on tumor cells were assessed with molecular biological and cellular approaches *in vitro* and *in vivo*. **Results**: *Albeit* broadly expressed in multiple normal tissues, *ZBTB28* is frequently downregulated in aero- and digestive carcinoma cell lines and primary tumors, and correlated with its promoter CpG methylation status. Further gain-of-function study showed that ZBTB28 functions as a tumor suppressor inhibiting carcinoma cell growth *in vitro* and *in vivo*, through inducing cell cycle arrest and apoptosis of tumor cells. ZBTB28 suppresses cell migration and invasion by reversing EMT and cell stemness. ZBTB28 transactivates TP53 expression, through binding to the p53 promoter in competition with BCL6, while *BCL6* itself was also found to be a direct target repressed by ZBTB28. **Conclusion**: Our results demonstrate that ZBTB28 functions as a tumor suppressor through competing with BCL6 for targeting p53 regulation. This newly identified ZBTB28/BCL6/p53 regulatory axis provides further molecular insight into carcinogenesis mechanisms and has implications in further improving BCL6-based anticancer therapy.

## Introduction

Cancer development is a multistage process in which cells gradually lose growth control and becomes transformed following oncogene activation and tumor suppressor inactivation 1. Oncoprotein and tumor suppressors have crucial but opposite roles in tumorigenesis, often as potential therapeutic targets. Their expression regulation by transcription factors is an important part of gene regulation. Studying the molecular mechanism of cancer-related transcription factors is of great significance to elucidate the mechanism of tumor development and progression.

Zinc-finger and BTB/POZ (Poxvirus and Zinc-finger) domain-containing protein (ZBTB) are family proteins of nuclear transcription factors 2. ZBTB proteins can directly regulate multiple target gene transcription through binding to their cis-regulatory elements 3. Some ZBTB genes can function as vital proto-oncogenes such as BCL6/ZBTB27; or important tumor suppressors such as HIC1/ZBTB29 4. BCL6 (B cell lymphoma 6)/ZBTB27, located at 3q27, contains BTB/POZ and zinc-finger motifs and functions as a transcriptional repressor 5. Its N-terminal BTB-POZ domain is a highly evolutionary conserved protein-protein interaction motif, consisting of around 130 amino acids. BCL6 forms a homodimer through interactions mediated by the BTB-POZ domain, and also heterodimerize to other BTB-POZ-containing proteins such as promyelocytic leukemia zinc finger (ZBTB16/PLZF) and Myc-interacting zinc finger protein-1 (Miz-1) 6. Altered BCL6 protein expression was also implicated in the pathogenesis of lymphomas and solid tumors, functioning as an oncogene by binding to hundreds of target genes 7, 8. Recent studies have shown BCL6 proto-oncogene emerging as a key therapeutic target 7, 9-11. However, the development of BCL6-targeted therapy has been seriously hampered due to its important normal physiological roles in several different cell types.

ZBTB28 (also called as BAZF, BCL6B or ZNF62), is a recently identified novel tumor suppressor candidate 12. Located at 17p13.1 13, ZBTB28 belongs to the POZ and Kruppel (POK) family of transcriptional repressors. Its predicted amino acid sequence indicated a BTB/POZ domain and five repeats of Kruppel-like zinc finger motif at the NH2-terminal and COOH-terminal region, respectively 14. The consensus binding sequence (CBS) of ZBTB28 is nearly the same as that of BCL6, indicating its important functions in oncogenesis 14. Notably, its amino acid sequence of zinc finger is 94% identical to BCL6 and contains a completely conserved middle portion. Thus, ZBTB28 may compete for the promoters of BCL6 target genes to perform its transcriptional inhibition function. More than 490 DNA binding sites and target genes of BCL6 have been identified in the human genome 15. It is unclear whether ZBTB28 will target these same target genes.

Human ZBTB28 mRNA is expressed ubiquitously in normal tissues, especially in heart and placenta 16. Interestingly, it is frequently down-regulated in various cancer tissues due to promoter methylation, including hepatocellular carcinoma (HCC) 17, gastric cancer (GsCa) 18 and colorectal cancer (CRC) 19. Low ZBTB28 expression in tumors is correlated with shorter overall survival in HCC and GsCa patients 12, 20, 21. ZBTB28 and BCL6 have been reported to be master regulators of self-renewal in spermatogonial stem cells (SSCs), and play pivotal roles in germinal center formation 22, 23. However, the role and mechanism of ZBTB28 in lung, nasopharyngeal (NPC) and esophageal squamous cell (ESCC) carcinomas remain to be determined.

Through CpG methylome study, we identified *ZBTB28* as a methylated target gene in carcinomas. This study aims to investigate its alterations and functions/ mechanisms in lung, nasopharyngeal and esophageal carcinomas. We found that promoter CpG methylation-mediated downregulation of *ZBTB28* was associated with poor prognosis and survival in lung cancer patients. We also found that ZBTB28 inhibits proliferation and invasion, as well as epithelial to mesenchymal transition (EMT) of lung, nasopharyngeal (NPC) and esophageal squamous cell (ESCC) carcinoma cells. Mechanistically, our data indicate that ZBTB28 directly targets p53 by competing with BCL6 and downregulating BCL6 expression. These results identify ZBTB28 as an important tumor suppressor gene (TSG), which may lead to novel strategies for the therapeutic control of BCL6 target in cancer treatment.

## Results

### *ZBTB28* is downregulated by CpG methylation in multiple carcinomas

**Figure 1:**
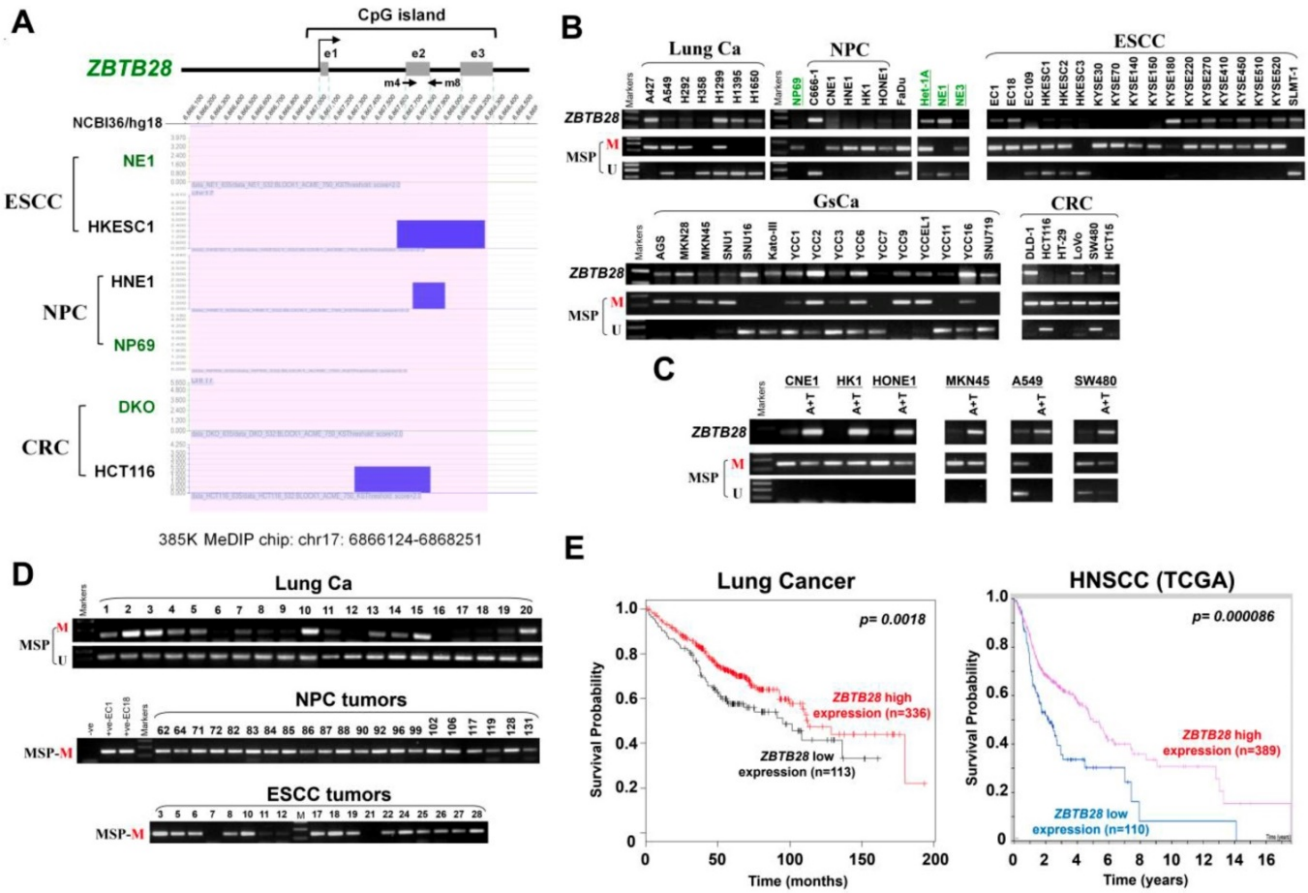
** Expression and methylation of *ZBTB28* in carcinoma cell lines. (A)** CpG methylome analysis demonstrated signal enrichment in *ZBTB28* promoter CGI in ESCC, NPC and colon cancer by MeDIP-Chip. Positive signal peaks (blue) are marked. **(B)*** ZBTB28* expression and methylation status in tumor cell lines. RNA integrity has been confirmed by *GAPDH* test shown in our previous publications. **(C)** Pharmacological demethylation of* ZBTB28* CGI by Aza (A), with or without TSA (T) and induction of expression. *ZBTB28* expression before and after drug treatment was determined by RT-PCR. Demethylation was confirmed by MSP. **(D)** Methylation status of *ZBTB28* promoter in lung, nasopharyngeal and esophageal tumor tissues. Representative data of *ZBTB28* methylation as shown. **(E)** Kaplan-Meier plots of the association between* ZBTB28* expression and survival in lung and HNSCC cancers. High *ZBTB28* expression had significantly longer survival. ESCC, esophageal squamous cell carcinoma; NPC, nasopharyngeal carcinoma; MSP, methylation-specific PCR; M, methylation; U, unmethylation.

We performed CpG methylome analysis to identified critical cancer genes in carcinomas and identified *ZBTB28* as a methylated target. Methylome data showed signal enrichment in *ZBTB28* promoter CpG island (CGI) in ESCC, NPC and colon cancer cell lines with or without DNMT knockout, while no signal was detected in immortalized normal epithelial cells (NE1 and NP69) and a colon cell line with genetic demethylation (DKO) (Figure 1A).

RT-PCR analysis showed that ZBTB28 is expressed widely in normal adult and fetal tissues (Figure S1A), but silenced or frequently downregulated in 5/8 lung, 5/6 NPC, 15/18 ESCC, 11/16 GsCa, and 5/6 colon cancer cell lines (Figure 1B). Further online bioinformatics analysis also revealed that ZBTB28 exhibits significant downregulation in multiple cancer types (Table 1). Meanwhile, *ZBTB28* promoter methylation was detected in almost all downregulated or silenced cell lines by MSP analysis (Figure 1B). The specificity of methylated primers was confirmed in not-bisulfite DNA (Figure S1B). Treatment with DNA methyltransferase inhibitor 5-aza-2'-deoxycytidine (Aza) and histone deacetylase inhibitor Trichostatin A (TSA) led to the restoration of *ZBTB28* expression, reduction of its methylation level, accompanied by an increase in unmethylated alleles (Figure 1C). Mutation analysis using public database showed that BCL6 is mostly amplified, whereas deletion or mutation of *ZBTB28* was detected in lung, head and neck squamous (HNSCC), ESCC and gastric carcinomas (Figure S2). These results suggest that *ZBTB28* silencing in carcinoma cells is mainly mediated by aberrant CpG methylation.

We further analyzed *ZBTB28* expression and methylation in primary tumors from Cancer Genome Atlas (TCGA) and GENT (Gene Expression across Normal and Tumor tissue) databases. Data showed significantly reduced *ZBTB28* expression and increased methylation in lung, HNSCC and esophageal cancer tissues, compared to their corresponding normal tissues (Figure S3A-B). Increased methylation of *ZBTB28* was significantly correlated with reduced ZBTB28 expression in lung cancer specimens from TCGA datasets (Figure S3C). Furthermore, ZBTB28 promoter methylation was detected in 87.5% (84/96) Lung, 100% (21/21) NPC, 67.5% (27/40) ESCC, 78.9% (30/38) GsCa, and 91.9% (34/37) colon primary tumors, but rarely in normal lung, nasal, esophagus, colon or gastric tissues (Figure 1D, Figure S1C,Figure S4). Moreover, *ZBTB28* high expression was significantly correlated with longer survival of patients with lung and HNSCC cancers (Figure 1E).

### ZBTB28 acts as a functional tumor suppressor in tumorigenesis

**Figure 2:**
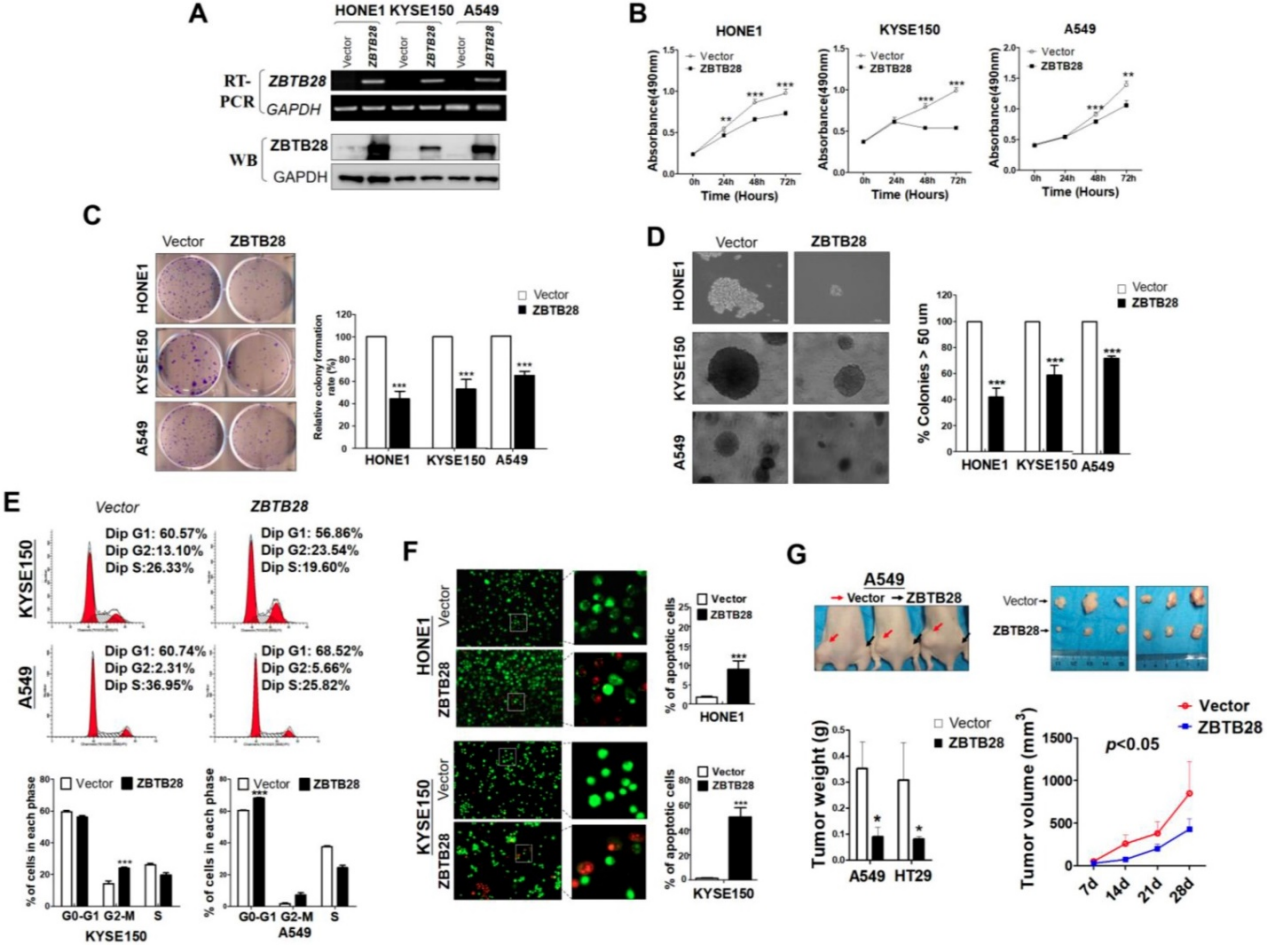
** ZBTB28 inhibits carcinoma cell growth *in vitro* and *in vivo*. (A)** Detection of *ZBTB28* overexpression by RT-PCR (upper panel) and Western blot (lower panel), with *GAPDH* as a control. Ectopic ZBTB28 expression inhibits carcinoma cell growth, as assessed by MTS **(B)**, colony formation **(C)** or soft agar assays **(D)**. Values are shown as mean±standard error from three independent experiments. **(E)** Cell cycle distribution of vector- or ZBTB28-transfected cells as determined by flow cytometry. Representative flow cytometry plots. Histograms of cell cycle alterations. **(F)** Induction of apoptosis detected by AO/EB assay. Histograms of apoptosis rate are shown at the right. Values are shown as mean±standard error from three independent experiments. **(G)** Xenografts before and after resection. Red and black arrows indicate empty vector control and ZBTB28-overexpressing tumors, respectively. Tumor weight measurements from empty vector control and ZBTB28-overexpressing tumors (n=5). Growth curve of xenograft tumors. Tumor volume was calculated from tumor length and width, measured twice per week.

Gain-of-function cell models were used to explore the specific role of ZBTB28 in tumorigenesis. The exogenous expression of ZBTB28 was verified by RT-PCR and Western blot (Figure 2A, Figure S5A). The effect of ZBTB28 on cell proliferation and viability was then examined by MTS assay and colony formation assay. Overexpression of ZBTB28 evidently inhibited the proliferation and colony forming of tumor cells (Figure 2B-D, Figure S5B-D). Flow cytometry analysis showed that ZBTB28-overexpressing cells consisted of a significantly higher proportion of sub-G1 or G2-M phase cells, and a reduced amount of S-phase cells, compared to control cells (Figure 2E, Figure S5E). ZBTB28 overexpression in tumor cells a promoted early apoptotic and late apoptotic of cells (Figure 2F, Figure S5F).

ZBTB28 anti-tumor ability was further examined in two (A549 and HT29) xenograft tumor models. The mean weight and volume of tumors were significantly lighter and smaller in mice that received ZBTB28-transfected cells compared to those that received cells transfected with a control vector (Figure 2G, Figure S5G). Immunohistochemistry (IHC) analyses showed that PCNA expression and the proliferation index in nude mice tissues were significantly decreased (Figure S6A-B). Collectively, these data indicate that ZBTB28 is able to significantly impede tumor growth, supporting the role of ZBTB28 as a functional tumor suppressor in human cancers.

### ZBTB28 suppresses cell migration and invasion by reversing EMT and cell stemness

**Figure 3:**
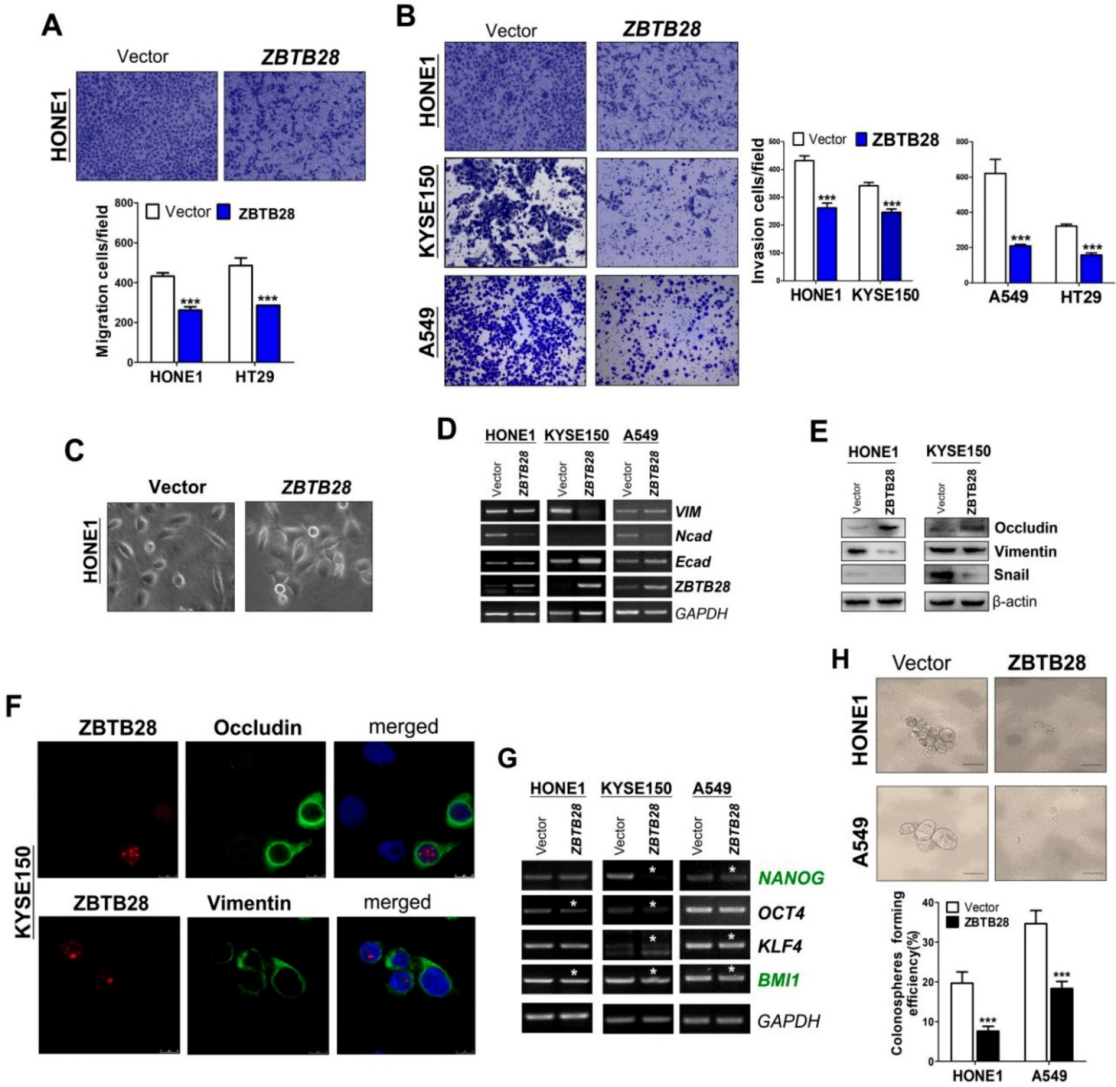
** ZBTB28 expression inhibits the migration and invasion of carcinoma cells through reversing EMT and disrupting stemness. (A)** Representative transwell assay. Pictures were taken at 24 h after seeding (magnification, 200x). **(B)** Migrating cells were counted in seven representative high power fields per transwell. Values are shown as mean±standard deviation from three independent experiments in triplicate, *p<0.05. ZBTB28 expression reverses EMT and disrupts stemness. **(C)** Morphological changes of cells transfected with ZBTB28 or empty vector, obtained by phase contrast microscopy. Original magnification, 400x. Expression of EMT markers in ZBTB28-expressing carcinoma cells, as determined by qRT-PCR **(D)** and Western blot **(E)**. GAPDH or actin was used as a control. **(F)** Expression and location of EMT markers in KYSE150 cells as detected by confocal microscopy. Original magnification, 400x. **(G)** Ectopic expression of ZBTB28 in carcinoma cells disrupt the expression of stemness markers. **(H)** The spheroid-forming assay showed that ZBTB28 lowered the spheroid-forming rates of A549 and HONE1 tumor cells.

To explore the role of ZBTB28 in metastasis, cells with different metastatic capacity were selected. We found that ZBTB28 overexpression resulted in decreased cell motility and invasiveness, compared to control cells (Figure 3A-B, Figure S7A). It has been well established that EMT is the critical step of invasion and metastasis. Our results showed that ZBTB28 overexpression resulted in a partial morphological change from scattered growth structures to tightly packed colonies, indicating that ZBTB28 is able to reverse EMT (Figure 3C). We found that ectopic ZBTB28 upregulated epithelial markers E-cadherin or occludin expression, downregulated mesenchymal markers N-cadherin and snail level in HONE1 and KYSE150 cells (Figure 3D-F, Figure S7B). EMT not only involves cellular motility but also confers self-renewal properties of tumor cells. We thus examined the change in cancer stem cells (CSC) biomarkers at the mRNA level. Results showed that ZBTB28 inhibited *NANOG*, *BMI1*, *OCT4*,* KLF4*, *TIP30* and *STAT3* mRNA expression in tumor cells (Figure 3G). The spheroid-forming assay also showed that ZBTB28 lowered the spheroid-forming rates of multiple tumor cells (Figure 3H). Collectively, our data suggest that ZBTB28 inhibits EMT and cell stemness in tumor cells.

### ZBTB28 and BCL6 inhibit each other's transcription

**Figure 4:**
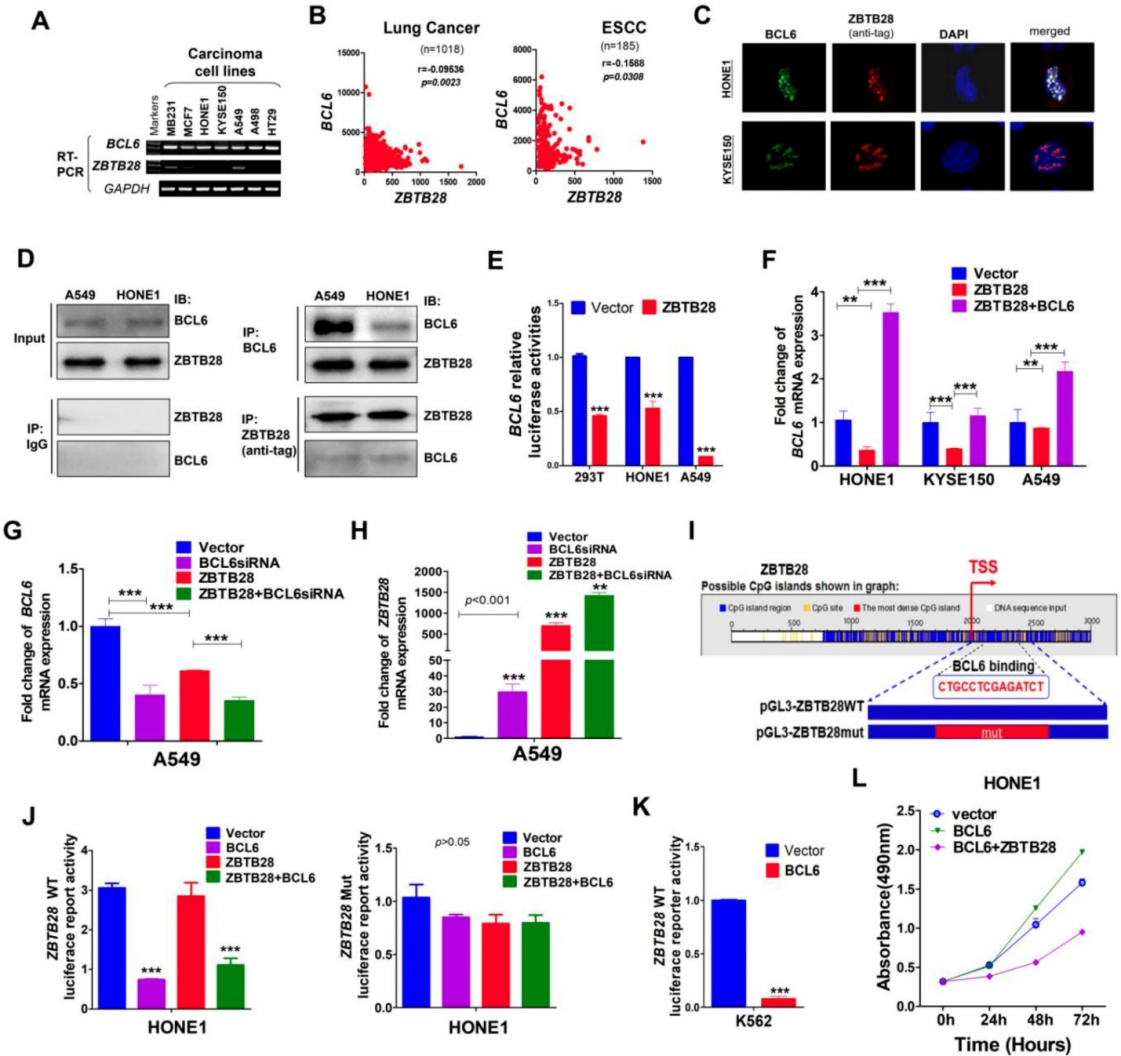
** ZBTB28 and BCL6 inhibit and depend on each other. (A)** ZBTB28 and BCL6 expression in multiple carcinoma cells. **(B)** Inverse association of ZBTB28 and BCL6 expression in lung cancer and ESCC, obtained from TCGA cancer dataset, accessed through cBioPortal (www.cbioportal.org). **(C)** Co-localization of BCL6 and ZBTB28 as determined using confocal microscopy. **(D)** Co-IP outcome revealing the binding between BCL6 and ZBTB28. Anti-HA (#ab1424, Abcam), BCL6 (sc-7388; Santa Cruz, Germany) or IgG (#2729, Cell Signaling Technology) antibodies were used. **(E)** ZBTB28 inhibits *BCL6* promoter activities, as detected by dual luciferase reporter system. **(F)** qRT-PCR results of *BCL6* mRNA expression in HONE1, KYSE150, and A549 cells. Cells were transfected with vector and ZBTB28 with or without BCL6. **(G)** qRT-PCR of *BCL6* mRNA in A549 underwent with vector, BCL6 siRNA, and ZBTB28 with or without BCL6 siRNA. **(H)** Knock-down BCL6 by siRNA increased *ZBTB28* mRNA expression levels. **(I)** Structure of wild type and mutant ZBTB28 reporter plasmid. **(J)** Promoter luciferase activity of wild-type and mutant ZBTB28 in HONE1 cells. Cells were transfected with BCL6, or ZBTB28 or both plasmids. **(K)** BCL6 inhibits *ZBTB28* reporter activity in BCL6-null K562 cells. **(L)** The interdependent role of ZBTB28 and BCL6 was detected using MTS assay in a tumor cell line which lack of ZBTB28 expression.

Protein-protein association networks predicted an interaction between ZBTB28 and BCL6 (Figure S8A-B). Our RT-PCR analysis showed that *BCL6* was expressed in carcinoma cell lines with silenced *ZBTB28* (Figure 4A). Meanwhile, data from TCGA online database confirmed an inverse relationship between *ZBTB28* and *BCL6* expression in lung and ESCC carcinomas (Figure 4B). Immunofluorescence assay demonstrated that ZBTB28 was co-localized with BCL6 in nuclei of carcinoma cells (Figure 4C). In addition, Co-IP results showed that ZBTB28 could form a complex with BCL6 (Figure 4D). Therefore, ZBTB28 and BCL6 may co-regulate gene expression in heterodimer form.

We then tested whether there is any inverse functional correlation between *ZBTB28* and *BCL6* expression using constructed luciferase reporters containing *BCL6* or *ZBTB28* promoters. We found that ectopic *ZBTB28* expression did inhibit luciferase reporter activities of *BCL6*-promoter and *BCL6* mRNA expression levels in both 293T and carcinoma (HONE1, A549) cells (Figure 4E-F).

We further determined the effect of BCL6 on *ZBTB28* expression. Knock-down of *BCL6* with siRNA upregulated ZBTB28 mRNA level in A549 cells (Figure 4G-H). On the other hand, we found putative BCL6 transcription factor-binding sites (TFBSs) in ZBTB28 promoter from Jaspar (http://jaspar.genereg.net/) and constructed the corresponding ZBTB28 wild-type or mutant reporter plasmids (Figure 4I). The results showed that exogenous expression of *BCL6* significantly inhibited the luciferase reporter activity of *ZBTB28* wild-type promoter construct, but not the mutant promoter (Figure 4J). The consistent results were further confirmed in the K562 cell line with no *BCL6* expression (Figure 4K) 24. These results indicate that ZBTB28 and BCL6 downregulate each other's RNA expression at the transcriptional level.

Additionally, we examined further whether *ZBTB28* expression antagonizes the cancer-promoting activity of BCL6 in carcinoma cells. As expected, BCL6 promoted the growth of HONE1 carcinoma cells that lacked ZBTB28 expression. When ZBTB28 was ectopically introduced, it strongly inhibited HONE1 cell growth even when BCL6 was present (Figure 4L). These results demonstrate that the anti- carcinogenic activity of ZBTB28 can even override the cancer-promoting function of BCL6.

### ZBTB28 directly promotes p53 transcription

**Figure 5:**
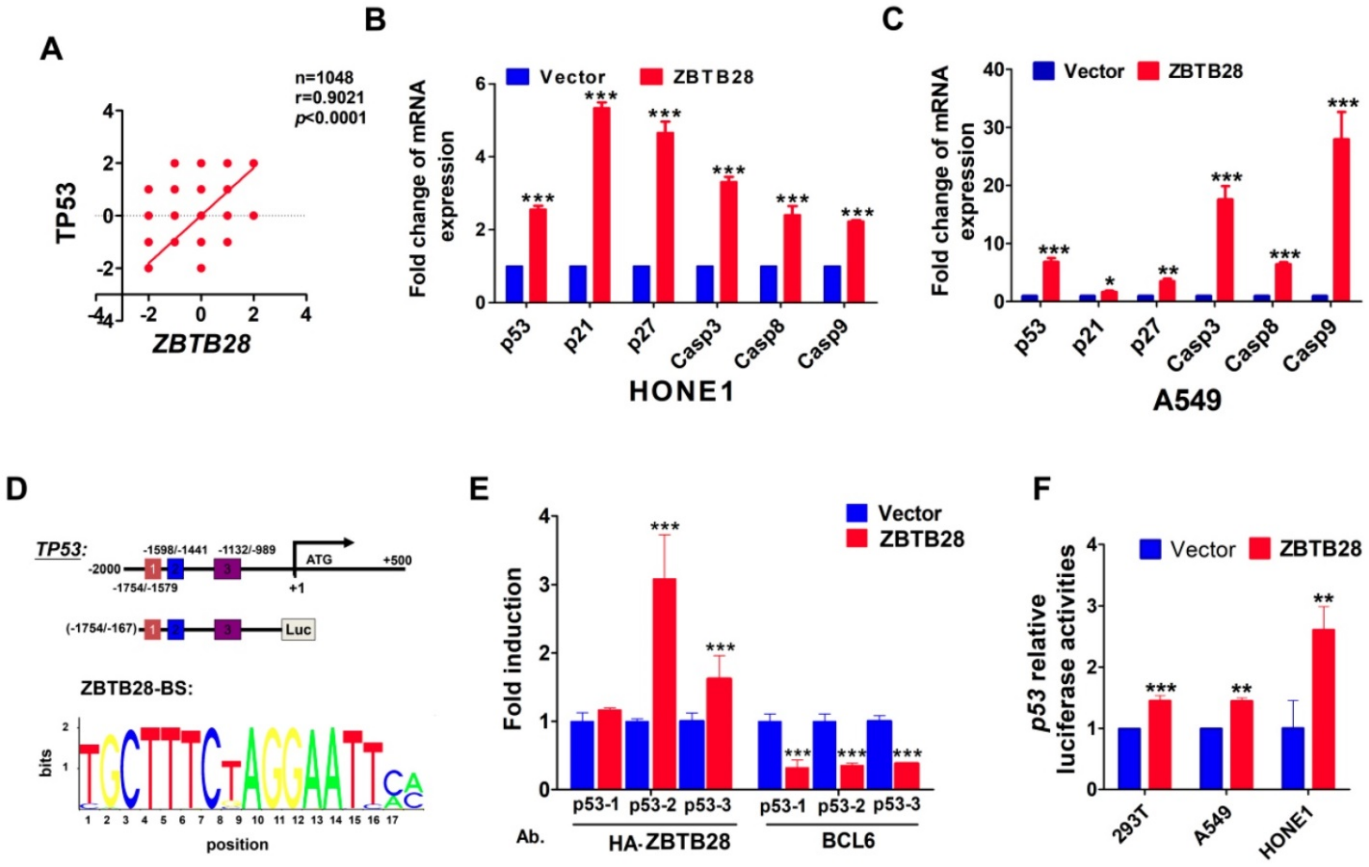
** ZBTB28 directly promotes p53 transcription in p53-wt cell lines. (A)** Data from the TCGA online database confirmed a positive relationship between ZBTB28 and p53 expression in tumor cells. **(B)** The expression of p53 and its downstream target genes as detected by qRT-PCR in ZBTB28-overexpressing and control cells. **(C)** The expression of p53 and its downstream target genes as detected by qRT-PCR in A549 cells with ZBTB28 overexpression (* *p*<0.05, ** *p*<0.01, *** *p*<0.001, vector vs ZBTB28). **(D)** Locations of ChIP PCR primers (for segment 1, 2 and 3) at the p53 promoter and structure of the p53 promoter-luciferase reporter construct. The bottom panel is the consensus binding sequence motif of ZBTB28. **(E)** ChIP analysis with HA-tag or BCL6 antibody, for ZBTB28- or BLC6 binding to the p53 promoter, in HONE1 cells. ChIP with a mouse IgG antibody was used as a negative control. ZBTB28 competes for BLC6 for its binding to the p53 promoter. **(F)** ZBTB28 activates the p53 promoter activity, detected using a dual luciferase reporter system.

In view of the highly similar binding domain of BCL6 and ZBTB28 (Figure S8C), we speculated that ZBTB28 might also directly regulate p53 transcription. We firstly searched for putative BCL6 transcription factor-binding sites (TFBSs) in p53 promoter from Jaspar (http://jaspar.genereg.net/). Three TFBSs for BCL6 (from -1708--1695, -1475- -1462 and -977--964) were identified in the p53 promoter. Data from the TCGA online database confirmed a positive relationship between ZBTB28 and p53 expression in tumor cells (Figure 5A). qRT-PCR showed that ZBTB28 did increase the mRNA levels of p53, and also upregulated p21, p27 and caspases (downstream targets of p53) levels in carcinoma cells (Figure 5B). Furthermore, knocking down BCL6 could decrease the expression of p53 and p53-targeted genes induced by ZBTB28 ectopic expression (Figure 5C, Figure S8D).

We further examined the binding of ZBTB28 to the previously reported BCL6-binding sites at the p53 promoter, using ChIP assays with chromatin isolated from HONE1 cells and antibodies against HA-ZBTB28 or BCL6. Results showed that ectopic ZBTB28 expression resulted in sufficient enrichment of ZBTB28 at the predicted region (binding site 2 and 3) but significantly decreased BCL6 enrichment at the same region (Figure 5D-E), while ChIP using a control IgG antibody showed no significant enrichment over the entire surveyed region. Luciferase reporter assay was performed to further determine the direct effect of ZBTB28 on the p53 promoter. Results showed that ZBTB28 expression significantly activated p53 promoter activities in 293T and tumor cells (Figure 5F). Collectively, we found, for the first time, that the ZBTB28 transcription factor activates p53 transcription and is involved in p53 signaling regulation in carcinoma cells.

### ZBTB28 inhibits several oncogenic signaling pathways in carcinoma cells

**Figure 6:**
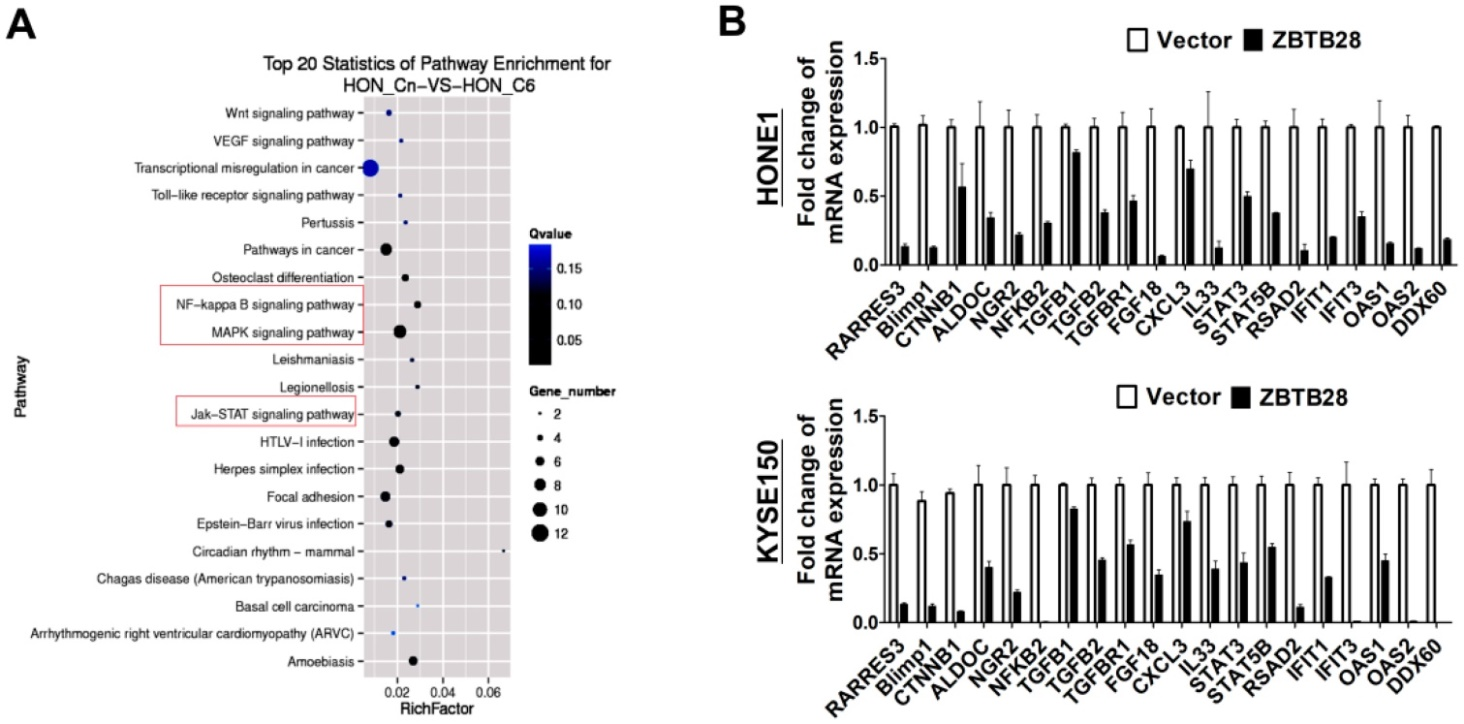
** ZBTB28 expression inhibits multiple oncogenic signaling in tumor cells**. **(A)** Differentially expressed genes from RNA sequencing analyses in tumor cells expressing ZBTB28 or empty vector. **(B)** Differential expression of identified ZBTB28-affected genes was confirmed by qRT-PCR in HONE1 and KYSE150 cells.

To gain a more comprehensive understanding of the molecular mechanisms by which ZBTB28 exerts its anti-tumor efficiency, we identified the differentially expressed target genes of ZBTB28 through RNA sequencing (RNA-seq) analysis. To improve the reproducibility of this analysis, we employed global transcriptional profiling of RNA isolated from three separate cell cultures of KYSE150and HONE1. RNA-seq analysis identified 3028 differentially expressed genes in KYSE150-expressing ZBTB28 cells (*p* < 0.05, FDR< 0.10) (Figure 6A, Figure S9). Dysregulated gene pathways were mainly involved in cell proliferation and apoptosis. Specifically, alterations in NF-κB, MAPK and JAK-STAT signaling pathways contributed to significant differences in expression profiles in carcinoma cells (Figure 6B). These data suggest the regulation of key oncogenic signaling pathways by ZBTB28 in tumorigenesis.

## Discussion

The major causes of poor survival of cancer patients are due to late diagnosis and high recurrence. Thus, identification of carcinogenetic mechanisms and developing efficient therapeutic targets are essential. Transcription factors play important roles in cancer occurrence, development and metastasis, as their dysregulation related closely to cell proliferation, apoptosis, invasion and angiogenesis. Transcription factors are the subject of many recent investigations, as common targets for developing new antitumor drugs 25-27, Understanding the functions and mechanisms of transcription factors has the potential to discover the new strategy of targeted treatments and prevention towards cancers.

ZBTB28 is a novel BCL6-homologous gene. The zinc finger motifs and the middle portion of the gene are highly identical between ZBTB28 and BCL6. Structurally, the biochemical character of ZBTB28 is similar to that of BCL6. Our study demonstrates that ZBTB28 was downregulated in multiple tumors and cell lines, associated with its promoter methylation. Ectopic ZBTB28 expression not only suppressed the proliferation, migration, and invasion, and promoted apoptosis of several carcinoma cells, but also inhibited xenograft tumor growth.

It has been reported that ZBTB28 can inhibit tumorigenesis by promoting p53 expression in colon and hepatocellular cancers 19, 21. Previous studies also showed that BCL6 inhibits p53 transcription by its POZ domain 28. BCL6 has been identified as a primary target of p53 29, and it can also regulate numerous other tumor suppressor genes and oncogenes, thereby promoting the development and progression of tumors 11. The vital role of BCL6 in the p53 pathway and other tumor-associated pathways makes it a novel therapeutic target for cancer therapy 7, 10, 11, 30, 31.

The role of BCL6 as a proto-oncogene is well established 30, 32-35. Researchers have been using RNA interference or soluble inhibitor to silence BCL6 to treat cancers *in vitro* or in an animal model, especially diffuse large B-cell lymphomas with aberrant BCL6 expression. Unfortunately, endothelial sprouting and branching were significantly enhanced when inhibitor 79-6 was applied to decrease tumor angiogenesis in the mouse model 30, 32-36. Interestingly, Ohnuki et al. found that ZBTB28 can promote the degradation of CBF1 through the CUL3 E3-ligase complex, ultimately supporting angiogenic sprouting 37. It remains unclear whether BCL6 effects ZBTB28 expression in order to promote angiogenesis, as it is difficult to manipulate the balance of BCL6 in a normal physiological state versus a carcinogenic state. Because BCL6 plays an important role under normal physiological conditions, particularly in the autoimmune system, long-term or excessive inhibition of BCL6 activity is harmful to the growth of normal cells. It is therefore important to determine the mechanisms of BCL6 and ZBTB28 interaction.

Numerous relevant researches has confirmed that many of BTB-ZF proteins share a BTB domain at the N-terminus and multiple copies of C2H2 zinc finger domains at the C-terminus. The Zinc finger domain usually mediates DNA binding, while the BTB domain promotes protein-protein interactions and co-factor recruitment. These BTB domains have been shown to form homodimers which raise DNA binding affinity and specificity for transcription factors. Some BTB pairs also are able to form heterodimers, such as BCL6 with Miz1, Miz1 with NAC1, Miz1 with ZBTB4, ZBTB32 with PLZF, ZBTB7A and PLZF 6, 38-41. BCL6 formed heterodimers with Miz1 and was recruited to the p21 promoter region, where there was a binding site for Miz1 but not BCL6 6. BCL6 and PLZF share many similar functional roles. They were co-expressed and found to co-localize in nuclear dots in many cell lines 39. Here, we demonstrate that knock-down of BCL6 with siRNA not only increased the mRNA levels of p53 and p21 but upregulated ZBTB28 levels in multiple cell lines. On the other hand, BCL6 levels decreased, but p53 levels increased when ZBTB28 was overexpressed in 293T and other cancer cells. Our results confirmed that ZBTB28 and BCL6 are co-expressed and co-localized in nuclear dots in many cell lines, as well as forming a complex. Therefore, ZBTB28 and BCL6 may co-regulate gene expression in heterodimer form.

Our experiments also confirmed the inverse relationship between ZBTB28 and BCL6 in multiple cancer cells. Interestingly, BCL6 is essential for the function of ZBTB28 as a tumor suppressor in cancer cells, however, BCL6 lost its cancer-promoting function when ZBTB28 expression was present.

These findings will make it possible to differentiate between the normal and carcinogenic activity of BCL6, and provides a theoretical basis for the design of new treatments that involve BCL6 inhibition. We also demonstrated that ZBTB28 is downregulated in various cancer cell lines by DNA methylation. Together, this suggests that ZBTB28 might have clinical utility as a cancer biomarker, or as the target of clinical intervention.

## Materials and Methods

### Cell lines, tumor samples and normal tissues

Multiple carcinoma cell lines (HONE1, A549, KYSE150, A498, HT29, *etc*) were obtained from the American Type Culture Collection (ATCC, Manassas, VA) or collaborators, and routinely maintained in RPMI 1640 media (Gibco-BRL, Karlsruhe, Germany) as recommended by ATCC. HCT116 DNMT1^-^/^-^DNMT3B^-^/^-^ (DKO) cell line was gifted by Prof. Bert Vogelstein (Johns Hopkins, Baltimore, MD).

Tissue samples used include primary tumor tissues and paired surgical margin tissues, as well as normal tissues. Tissues were obtained from the First Affiliated Hospital of Chongqing Medical University. All samples were reviewed and subjected to histological diagnosis by pathologists to ensure that the percentage of tumor cells was over 70%. Clinical and pathological information was then collected. This study was authorized by Institutional Ethics Committees of the First Affiliated Hospital of Chongqing Medical University (Approval notice: # 2016-75) and abided by the Declaration of Helsinki.

### CpG methylome analysis

We performed CpG methylome analysis by methylated DNA Immunoprecipitation (MeDIP) using NimbleGen 385K CpG Island Plus Promoter Array as described previously 42.

### Reverse transcription (RT)-PCR and real-time PCR

Genomic DNA and total RNA were isolated from cell lines and tissues. TRI Reagent® (Molecular Research Center, Cincinnati, OH) was used to isolate total RNA and DNAzol® reagent (Invitrogen, Rockville, MD) or the QIAamp® DNA Mini Kit (Qiagen, Hilden, Germany) was used to extract DNA.

RT-polymerase chain reaction (RT-PCR) was performed as previously described. *GAPDH* was amplified as a control. Samples were assayed in a 10 µl reaction mixture containing 2 µl of cDNA. The primer sequences are listed in Table 2. RT-PCR was carried out using Go-Taq (Promega, Madison, WI). Reactions were performed under the following conditions: 32 cycles for *ZBTB28* and 23 cycles for *GAPDH*. Real-time PCR was performed according to the instrument manual (HT7500 System; Applied Biosystems, Foster, USA). The primer sequences are listed in Table 3. The mean expression level of ZBTB28 in paired surgical margin tissues was considered as 1, and the relative expression levels of ZBTB28 in tissues were standardized to β-actin levels. An online cancer database (http://www.oncomine.org) was used to examine the mRNA expression of *ZBTB28* in normal versus tumor tissues. *P-values* for each group were calculated using a Student's t-test. Standardized normalization techniques and statistical calculations are provided on the Oncomine website, and as previously described 43.

### Bisulfite conversion and methylation-specific PCR (MSP)

Bisulfite conversion of DNA and methylation-specific PCR (MSP) were carried out as previously described 44. The methylation-specific primers and annealing temperatures are listed in Table 4. MSP analysis revealed no amplified products in any non-bisulfited DNA by MSP primers, thus it can be considered specific. AmpliTaq^®^-Gold DNA Polymerase (Applied Biosystems) was used for MSP, with 40 reaction cycles. PCR products were separated on a 2% agarose gel and photographed on a gel imaging system (Bio-RAD Gel Doc XR+, CA). To elucidate whether promoter CpG methylation might regulate the expression of *ZBTB28*, several tumor cell lines were treated with a demethylation agent and/or a histone deacetylation inhibitor, as we have previously described 45, 46.

### Construction of plasmids and stable cell lines

We generated multiple ZBTB28 and BCL6 expression vectors to be used for various purposes. To construct pcDNA-ZBTB28, the *ZBTB28* full-length gene with an HA tag was inserted into a pcDNA3.1(+) framework plasmid. The resultant plasmid was transformed into *E. coli* DH5a cells and sequenced. The recombinant plasmids pMyc-*ZBTB28* and Lv-*ZBTB28* were constructed by sub-cloning based on the pcDNA-HA-*ZBTB28* plasmid. To construct the pEGFPC1-BCL6 plasmid, the BCL6 full-length gene was inserted into pEGFPC1 at KpnI and BamHI sites.

Stably transfected pcDNA-ZBTB28 cells were obtained using G418 selection (300 μg/ml for HONE1, 700 ug/ml for KYSE150, 600 ug/ml for HT29, 400 ug/ml for A549). Total RNA and protein from stable cell lines were extracted using TRI Reagent® or Protein Extraction kit (Thermo Scientific, #23225), respectively. RNA was treated with DNase (Ambion, Austin, TX) to exclude plasmid contamination. Then, RT-PCR and western blotting were performed in order to measure the ectopic expression of *ZBTB28* prior to additional experiments.

### Cell proliferation assay and colony formation assays (CFA)

The proliferation of cells stably expressing *ZBTB28* was measured at 24, 48, and 72 h using the Cell Counting Kit-8 (CCK-8; Beyotime, Shanghai, China). Colony formation assays (CFA) were performed as previously described 47. Stable cell lines were cultured for 2 weeks at a density of 1 × 10^3^ cells/well. Surviving colonies (≥ 50 cells/colony) were stained and counted. All experiments were independently repeated in triplicate.

### The soft agar colony formation assay

The soft agar assay was used to confirm cellular anchorage-independent growth *in vitro*. Briefly, cells were plated in 6-well plates at 37ºC with 0.35% top agarose containing 1 × 10^3^ cells, and 1.2% bottom agarose harboring with corresponding medium (RPMI 1640), supplemented with 10% fetal bovine serum (FBS) in all agarose solutions. Cell colonies were photographed and counted under a 10× Leica microscope after three weeks of incubation. Each experiment was repeated three times.

### Spheroid-forming assays

Cells were cultured with serum-free DMEM/F12 (Invitrogen, Carlsbad, USA) containing 100 IU/ml penicillin, 100 μg/ml streptomycin, 0.4% bovine serum albumin (BSA), 4 μg/ml insulin (Invitrogen), 20 ng/ml recombinant human basic fibroblast growth factor (hrbFGF; Invitrogen), 20 ng/ml recombinant human epidermal growth factor (hrEGF; Invitrogen),and 2% B27 (Invitrogen). The number of tumor spheroid was counted under the microscope (Olympus, Tokyo, Japan) after 14 days of culture. The efficiency of spheroid formation was calculated as a fraction of the total number of spheroids generated by the number of live cells seeded, and multiplied by 100.

### Cell cycle and apoptosis analyses

To assess cell cycle, cells were cultured (1 × 10^6^ cells/well) for 48 h, then collected and analyzed using CELL Quest software (BD Biosciences, San Jose, CA), as previously described. For apoptosis analyses, cells were stained with acridine orange/ethidium bromide (AO/EB) and visualized under a fluorescence microscope (LEICA CTR4000B; Leica Microsystems, Buffalo Grove, IL, USA). Apoptotic rate (%) = (apoptotic cells/total cells) × 100%.

### Wound healing and Transwell^®^ assays for cell migration

Wound healing and Transwell^®^ assays were used to assess cell mobility. Stable cell lines were cultured in 6-well plates until confluent. A cellular wound was performed using a sterilized pipette tip, followed by washing of the cells twice, then further culturing for 12, 24 and 48 h in RPMI 1640 media containing 5% FBS. Images were obtained using a 10× objective lens at the indicated time periods. Transwell^®^ assays were carried out as described previously 47. Three independent experiments of each assay were repeated.

### Indirect immunofluorescence determinations

Cells were transiently transfected with *ZBTB28* and double stained according to a previously described protocol 43. The primary antibodies used were as follows: E-cadherin (#1702-1; Epitomics, Cambridge, MA), vimentin (#2707-1; Epitomics), HA (#3724, Cell Signaling Technology, Danvers, MA), and ZBTB28 (ab180084, Abcam). Anti-mouse IgG Alexa Fluor® 594 (Invitrogen) or anti-rabbit FITC (Dako, Carpinteria, CA) were used as secondary antibodies. DAPI (4′,6-diamidino-2-phenylindole) was used as a nuclear counterstain. Cells were then imaged using a confocal laser scanning microscope.

### Tumor xenograft model in nude mice

BALB/c nude mice (aged 4-6 weeks, weighing 18-22 g) were purchased and reared according to ethical guidelines by the Experimental Animal Center of Chongqing Medical University (CQMU), China. Stable *ZBTB28*-expressing cells or control cells (5 × 10^6^ cells in 0.1 ml PBS) were injected into the lower backs of nude mice (six mice per group). The longest and shortest diameters of tumors were measured using vernier calipers every 3 days for 30 days. Tumor volume (mm^3^) was calculated as follows: volume = length × width^2^ × 0.52.

### Co-immunoprecipitation (Co-IP) and Western blot

Co-IP assay was carried out to investigate the interaction of ZBTB28 and BCL6 protein. Briefly, stable ZBTB28-expressing cells HONE1 and A549 were transfected with BCL6 plasmids. Total proteins were extracted with ice-cold low salt lysis buffer, then, the cell lysate was incubated with anti-HA (#ab1424, Abcam), BCL6 (sc-7388; Santa Cruz, Germany) or IgG (#2729, Cell Signaling Technology) antibody overnight on a turning wheel at 4 °C. MilliporeSigma^Tm^PureProteome^Tm^ Protein A/G Mix Magnetic Bead System (#LSKAGAG10, Fisher Scientific, USA) was used to purify co-IP complex, the low salt lysis buffer used to wash the beads. The co-immunoprecipitation complex was analyzed by SDS-PAGE and Western blot.

Western blot was performed as our previously described protocol 48. All antibodies used for western blotting were as follows: ZBTB28 (ab180084; Abcam), BCL6 (sc-7388; Santa Cruz, Germany), active β-catenin (#05-665; Merck Millipore, Billerica, MD), β-catenin (#2677; Cell Signaling Technology), c-Myc (#1472-1; Epitomics), cyclin D1 (#1677-1; Epitomics), occludin (ab31721; Abcam), vimentin (#2707-1; Epitomics), E-cadherin (#1702-1; Epitomics), N-cadherin (ab98952; Abcam), β-actin (LK-ab008-100; Liankebio, China), and GAPDH (#AE082046; Beijing Biosynthesis Biotechnology, Beijing, China). A chemiluminescence kit was used to visualize protein bands (Amersham Pharmacia Biotech, Piscataway, NJ).

### Luciferase reporter assays

Reporter gene plasmids were generated using the pGL3/Basic plasmid as a framework, which expresses the firefly luciferase reporter. The promoter regions of genes were amplified by PCR and cloned into the pGL3/Basic plasmid using a seamless cloning kit (D7010S, Beyotime, Beijing, China), the pGL3-ZBTB28mut plasmid was synthetized by Genscript company(Nanjing, China), and was further confirmed by sequencing.

For ZBTB28 reporter assays, 293T, HONE1, or A549 cells were co-transfected with ZBTB28, pGL3-gene, and pRT-LK (80:1 ratio). The promoter-less pGL3/Basic vector was used as a negative control. Renilla luciferase was used as an internal control to assess transfection efficiency. At 24 h post-transfection, cells were lysed in 200 μl lysis buffer. For the luciferase read-out, 20 μl of the sample was detected using the Dual-luciferase reporter assay kit (Promega), followed by an additional 100 μl substrate. Light emission was quantified using a standard manual on the Luminometer (Infinite M200 PRO, Tecan, Austria). Five independent transfections were performed in each case.

### Chromatin Immunoprecipitation (ChIP) assay

Chromatin immunoprecipitation (ChIP) was performed according to the manual of the SimpleChIP® Enzymatic Chromatin IP Kit (#9003, Cell Signaling Technology). Cells were washed with ice-cold PBS devoid of Ca^2+^ & Mg^2+^ and supplemented with a protease inhibitor cocktail (PIC, P8340, Sigma-Aldrich, St. Louis, MO, USA). DNA and protein were cross-linked with 1% formaldehyde at 37°C for 10 min at room temperature. Reactions were terminated with 0.125 M glycine for 5 min. Cells were then lysed in lysis buffer (20 mM Tris-HCl, pH 8.0, 50 mM NaCl, 5 mM CaCl_2_, 1% Triton X-100, PIC) for 10 min, and micrococcal nuclease was added for 20 min at 37°C. Cells were then sonicated on ice for six sets of 10-sec pulses at 40% amplitude using an ultrasonic cell disruptor (JY88-IIN, Scientz, China) at an interval of 1 min, in order to generate 200-900bp DNA fragments. A 10 µl sample of the diluted chromatin lysates was removed to serve as an input sample. The diluted samples were incubated with HA antibody (#3274, Cell Signaling Technology), or BCL6 antibody (#14895, Cell Signaling Technology) overnight at 4°C, followed by capture with protein A/G magnetic beads (#9006, Cell Signaling Technology) for 2 h. Histone H3 antibody (#4620, Cell Signaling Technology) and Normal rabbit IgG (#2729, Cell Signaling Technology) were used as positive and negative controls, respectively. The complexes were precipitated, washed, and eluted as recommended. After DNA-protein cross-linkages were incubated with 6 µl of 5M NaCl and 2 µl of proteinase K at 65°C for 2 h, DNA was purified using spin columns for further use in PCR analyses. An equivalent volume of each sample was used as a template for PCR amplification. Specific primers are listed in Suppl. Table 2.

### Bioinformatics

Sequences of cDNA were analyzed using the NCBI Blast program (http://www.ncbi.nlm.nih.gov/BLAST/). Conserved regions were searched by multiple alignments of genomic sequences using ClustalW (http://www.ebi.ac.uk/Tools/clustalw2/index.html). Potential transcription factor binding sites were identified with MatInspector (http://www.genomatix.de//matinspector.html) and the Jaspar database (Jaspar.genereg.net).

### Statistical analyses

Statistics were analyzed using SPSS software, version 16 (SPSS, Chicago, IL, USA). A Student's t-test, Chi-squared test, or a Fisher's exact test was used. When *p*<0.05, data were considered to be statistically significant for all experiments.

## Supplementary Material

Supplementary figures.

## Figures and Tables

**Table 1:** ZBTB28 exhibits significant downregulation in multiple cancer types

Tissue type	Sample number	Median of expression intensity (log2)	*p* value
Lung	20	2.428	9.81E-9
Lung Adenocarcinoma	226	1.169	
Colon	79	0.002	4.55E-41
Colon Adenocarcinoma	212	-0.314	
Stomach	94	0.006	1.40E-5
Gastric Intestinal Type Adenocarcinoma	41	-0.097	
Liver	59	0.01	1.82E-17
Hepatocellular Carcinoma	97	-0.312	
Bladder	24	0.004	1.16E-7
Infiltrating Bladder Urothelial Carcinoma	99	-0.113	
Pancreas	39	1.115	1.18E-6
Pancreatic Ductal Adenocarcinoma	39	0.827	

**Table 2:** List of RT-PCR primers used in this study

PCR	Primer	Sequence (5'-3')	Product size (bp)	PCR Cycles	Annealing temperature (℃)
RT- PCR	*ZBTB28F*	CTACGTCCGCGAGTTCACTC	170bp	32	55
	*ZBTB28R*	CCCGGAAAATTGAATAGAAG			
	*BCL6F*	ATGAGTGTGACTGCCGCTTCTC	245bp		55
	*BCL6R*	GTAGGGCTTCTCTCCAGAGTGA			
	*VIMF*	TGCCAACCTTTACAGACCTA	390bp		55
	*VIMR*	CTCATCTCCCTCCTCACTCA			
	*EcadF*	CCTCCGTTTCTGGAATCCAA	282bp		55
	*EcadR*	GTTCTCTATCCAGAGGCTCT			
	*NcadF*	CAGGTTTGGAATGGGACAGT	480bp		55
	*NcadR*	TCCAGTAGGATCTCCGCCAC			
	*β-actinF*	TCCTGTGGCATCCACGAAACT	315bp	23	55
	*β-actinR*	GAAGCATTTGCGGTGGACGAT			

**Table 3:** List of qRT-PCR primers used in this study

PCR	Primer	Sequence (5'-3')	Product size (bp)	PCR Cycles	Annealing temperature (℃)
qRT- PCR	*RARRES3F*	ATGGCTACGTGATCCATCTG	151bp		60
	*RARRES3R*	AAGCTGTTGTTGACCCGATAG			
	*BlimpF*	AACCTGGCTGCGTGTCAGAAC	182bp		60
	*BlimpR*	CTCGGTTGCTTTAGACTGCTCTG			
	*CTNNB1F*	CTCAGTCCTTCACTCAAGAA	102bp		60
	*CTNNB1R*	CATCTAATGTCTCAGGGAACA			
	*ALDOCF*	CCATGAGACCCTCTACCAGA	128bp		60
	*ALDOCR*	TTTCTCCATCAGTCCCAGCTA			
	*NRG2F*	CAAGATCCTGTGCACTGACTG	146bp		60
	*NRG2R*	TGCCATCCTTGAACCAACGG			
	*NFKB2F*	AGATCTGTAACTACGAGGGACC	191bp		60
	*NFKB2R*	CTTCTTAGTCACATGCAGGACAC			
	*TGFB1F*	AATTGAGGGCTTTCGCCTTAG	87bp		60
	*TGFB1R*	CCGGTAGTGAACCCGTTGAT			
	*TGFB2F*	GACGAAGAGTACTACGCCAA	152bp		60
	*TGFB2R*	GCATCAAGGTACCCACAGAG			
	*TGFBR1F*	ACGGCGTTACAGTGTTTCTG	100bp		60
	*TGFBR1R*	CTTTGTCTGTGGTCTCTGTG			
	*FGF18F*	TGCATGAACCGCAAAGGCAA	200bp		60
	*FGF18R*	ATGAAATGCACGTCCTGCTG			
	*CXCL3F*	GTGGTCACTGAACTGCGCTG	127bp		60
	*CXCL3R*	TGAGTGTGGCTATGACTTCG			
	*IL33F*	GCAAAGTGGAAGAACACAGC	93bp		60
	*IL33R*	CATAAAGTACATGGGGCAAACT			
	*STAT3F*	ACCAAGCGAGGACTGAGCA	151bp		60
	*STAT3R*	CCAGCCAGACCCAGAAGG			
	*STAT5BF*	GATCAAGCTGGGGCACTATG	161bp		60
	*STAT5BR*	ACATGGCATCAGCAAGGCTT			
	*RSAD2F*	CGTGAGCATCGTGAGCAAT	188bp		60
	*RSAD2R*	AATCCCTACACCACCTCCTCA			
	*IFIT1F*	AGCCATTTTCTTTGCTTCCC	205bp		60
	*IFIT1R*	ACAGAGCCTTTTCTTCGGTA			
	*IFIT3F*	GGAAACTACGCCTGGGTC	180bp		60
	*IFIT3R*	CACCTTCGCCCTTTCATT			
	*OAS1F*	CTGACCTGGTTGTCTTCC	137bp		60
	*OAS1R*	GACCTCAAACTTCACGGA			
	*OAS2F*	TGAAGCCCTACGAAGAAT	176bp		60
	*OAS2R*	ACTGAAGAAGAGGACAAGG			
	*DDX60F*	GCAAAACCTATGCCTCCTAC	186bp		60
	*DDX60R*	GACGATACTCCCTGGTGAAA			
	*EcadF*	TACACTGCCCAGGAGCCAGA	103bp		60
	*EcadR*	TGGCACCAGTGTCCGGATTA			
	*NcadF*	CGAATGGATGAAAGACCCATCC	174bp		60
	*NcadR*	GGAGCCACTGCCTTCATAGTCAA			
	*β-actinF1*	GTCTTCCCCTCCATCGTG	113bp		60
	*β-actinR1*	AGGGTGAGGATGCCTCTCTT			

**Table 4:** List of MSP and Chip-PCR primers used in this study

PCR	Primer	Sequence (5'-3')	Product size (bp)	PCR Cycles	Annealing temperature (℃)
**MSP**	*ZBTB28m4*	GTCGTTATGGGTTTTTTCGTC	142bp	40 40	60
	*ZBTB28m8*	CGCCAACCAACAACGTAACG			
	*ZBTB28u4*	GTGTTGTTATGGGTTTTTTTGTT	145bp		58
	*ZBTB28u8*	CCACCAACCAACAACAACATAACA			
**Chip-PCR**					
	*Chipp53-1*	CATGATTGCACCACTGTACC	176bp		60
		CCAGGCTGGTGTGGAACTC			
	*Chipp53-2*	GGAGTTCCACACCAGCCTG	157bp		60
		CTCACTGCAACCTCTGCCTT			
	*Chipp53-3*	CCTGCCCTTAGGAAGTGTATA	143bp		60
		CTCCTTCCCGTGCAGACTTT			
