# Supplementary material for: Tumor suppressive BTB/POZ zinc-finger protein ZBTB28 inhibits oncogenic BCL6/ZBTB27 signaling to maintain p53 transcription in multiple carcinogenesis
Source: Theranostics. 2019 Oct 18;9(26):8182–95. doi: 10.7150/thno.34983 (PMC6857043; doi:10.7150/thno.34983)
Supplement: Supplementary file 1 — Supplementary figures. [file thnov09p8182s1.pdf]

Supplementary figure legends

Figure S1. (A) ZBTB28 expression in human normal adult tissues by semi-quantitative RT-PCR, *GAPDH* same as previous studies. (B) The specificity of MSP primers for methylated promoter alleles was tested using not-bisulfited DNA. (C) *ZBTB28* methylation detection in normal lung and nasal tissues. M, methylation; U, unmethylation.

Figure S2. Somatic alterations of *ZBTB28* and *BCL6* from Public database. Database query was based on amplification, deletion, and mutation alterations of the gene, using data available at http://www.cbioportal.org. Obviously, amplification of *BCL6* is common in some cancers, but very rare for *ZBTB28*. The major genetic alterations of *ZBTB28* are deletions and mutations, although they are still rare in multiple cancers.

Figure S3. Expression and methylation status of ZBTB28 in multiple tumor tissues. (A) Quantitative reverse transcription-PCR (qRT-PCR) analysis of ZBTB28 mRNA expression in multiple tumor tissue samples and paired adjacent normal tissues, data available at http://mgrc.kribb.re.kr/GENT/overview.php. (B) Methylation of the *ZBTB28* promoter in normal and tumor tissues from MethHC database. (C) Downregulation and promoter methylation of ZBTB28 in lung cancer tissue samples and paired non-cancerous samples from Cancer Genome Atlas (TCGA), data accessed via MethHC.

Figure S4. Methylation status of *ZBTB28* promoter in colon and gastric tumor tissues and normal tissues. Representative data of *ZBTB28* methylation in (A) tumor and (B) normal tissues. M: methylated; U: unmethylated.

Figure S5. ZBTB28 functions as a tumor suppressor in colon tumor cells. (A) ZBTB28 expression at mRNA and protein levels was examined by

semi-RT-PCR (upper panel) and Western blot (lower panel) in HT29 cells, with GAPDH as a control. (B) Ectopic ZBTB28 expression inhibits HT29 cell growth, as assessed by MTS (B), colony formation (C) or soft agar assays (D). Values are shown as mean±standard error from three independent experiments. (E) Cell cycle distribution of vector- or ZBTB28-transfected HT29 cells as determined by flow cytometry. Representative flow cytometry plots. Histograms of cell cycle alterations. (F) Induction of apoptosis detected in ZBTB28-expressing HT29 cells by AO/EB assay. Histograms of apoptosis rate are shown at the bottom. Values are shown as mean±standard error from three independent experiments. (G) ZBTB28 inhibits tumor growth of HT29 xenograft *in vivo*. Red and black arrows indicate empty vector control and ZBTB28-overexpressing tumors, respectively. Tumor weight measurements from empty vector control and ZBTB28-overexpressing tumors (n=5). Growth curve of xenograft tumors. Tumor volume was calculated from tumor length and width, measured twice per week.

Figure S6. ZBTB28 inhibits tumor growth of A549 xenograft *in vivo*. (A) Representative images of PCNA immunohistochemistry staining and (B) score of proliferation index.

Figure S7. ZBTB28 inhibits tumor cell migration and EMT. (A) Representative wound healing assay. Photographs were taken at 0 and 24 h of ZBTB28-transfected HONE1 and KYSE150 tumor cells. (B) Expression and location of Occludin and Vimentin markers in HONE1 cells as detected by confocal microscopy. Original magnification, 400x.

Figure S8. (A-B) Protein-protein association networks predicted an interaction between ZBTB28 and BCL6. (C) Predicted domain structure of ZBTB28 and BCL6 proteins showing the BTB protein-protein interaction domain and Zinc-finger domain (COSMIC, http://cancer.sanger.ac.uk/cosmic). Binding

protein motif prediction showed a highly homologous binding domain between BCL6 and ZBTB28. (D) The expression of p53 and its downstream target genes as detected by qRT-PCR in A549 cells with ZBTB28 overexpression and BCL6 knockdown (** $p<0.01$, *** $p<0.001$, ZBTB28 vs ZBTB28+siBCL6).

Figure S9. RNA sequencing analyses reveals differentially expressed genes in KYSE150 tumor cells expressing ZBTB28.

Figure S1

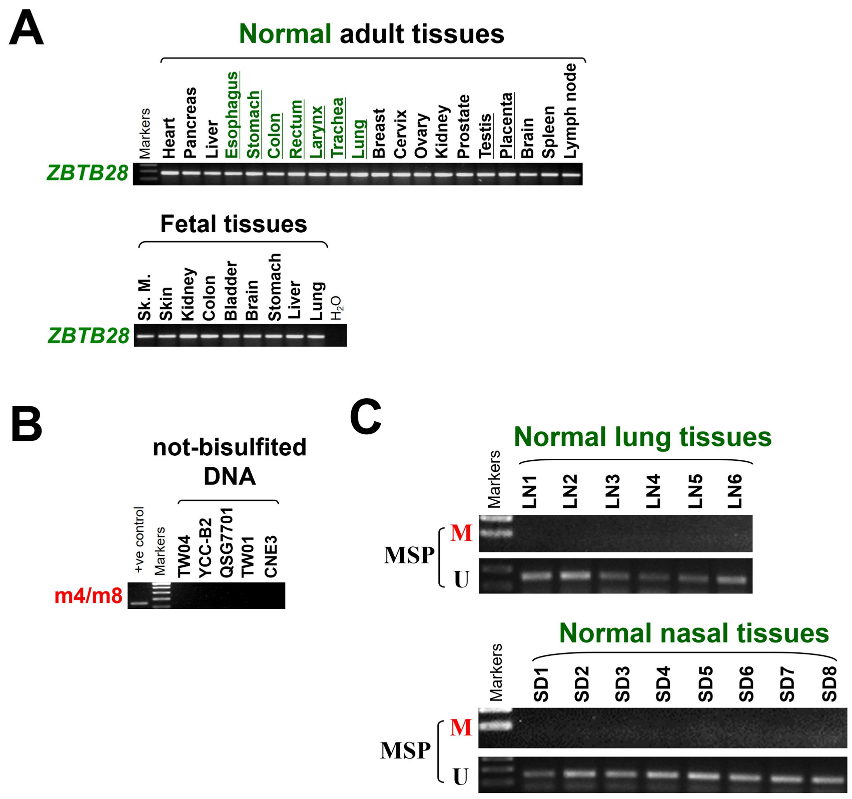
A
Normal adult tissues
Markers
Heart
Pancreas
Liver
Esophagus
Stomach
Colon
Rectum
Larynx
Trachea
Lung
Breast
Cervix
Ovary
Kidney
Prostate
Testis
Placenta
Brain
Spleen
Lymph node
ZBTB28
Fetal tissues
Sk. M.
Skin
Kidney
Colon
Bladder
Brain
Stomach
Liver
Lung
H2O
ZBTB28
B
not-bisulfited DNA
+ve control
Markers
TW04
YCC-B2
QSG7701
TW01
CNE3
m4/m8
C
Normal lung tissues
Markers
LN1
LN2
LN3
LN4
LN5
LN6
MSP
M
U
Normal nasal tissues
Markers
SD1
SD2
SD3
SD4
SD5
SD6
SD7
SD8
MSP
M
U

Figure S2

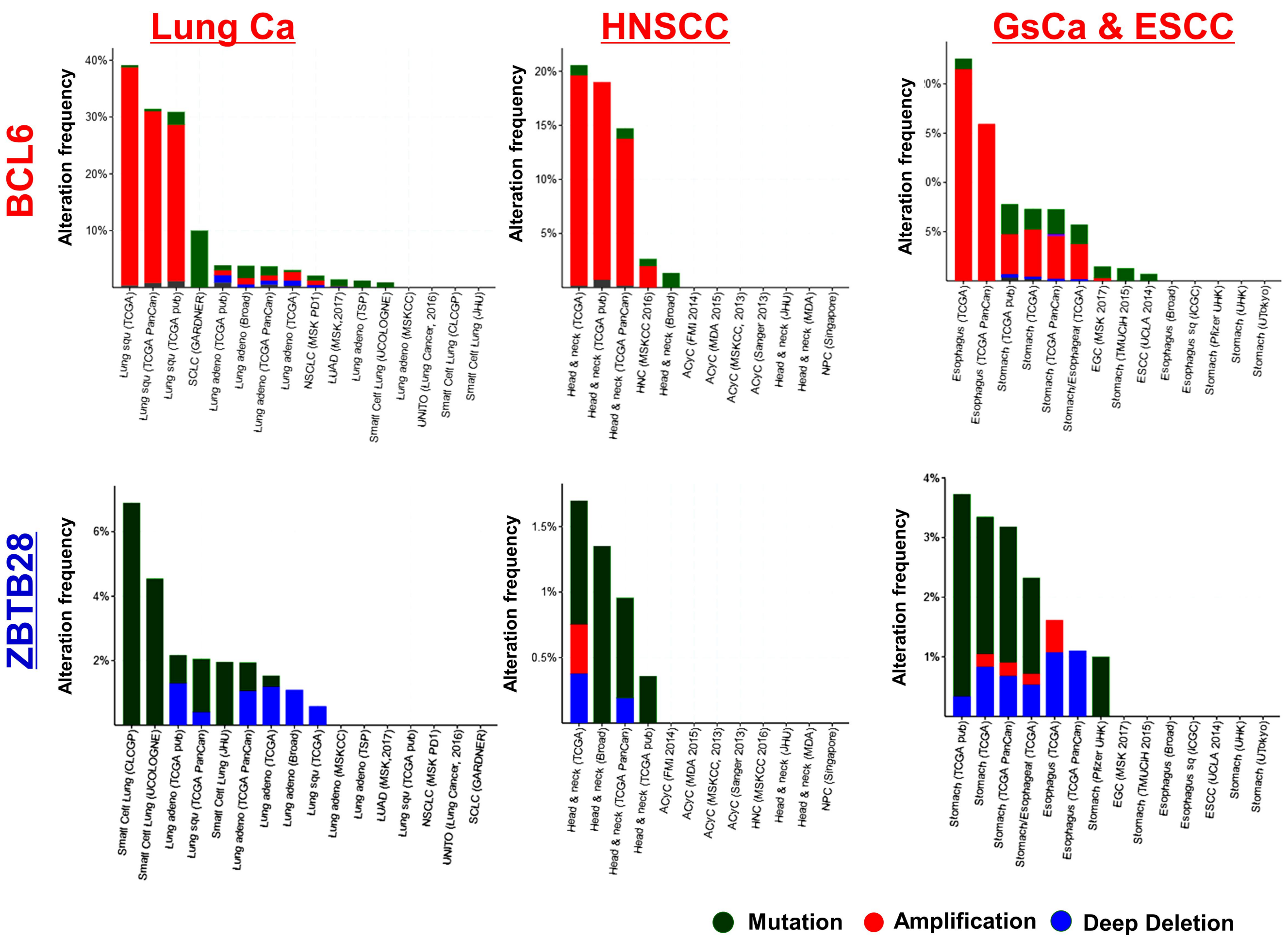

Lung Ca
HNSCC
GsCa & ESCC
BCL6
ZBTB28
Alteration frequency
Mutation
Amplification
Deep Deletion

Figure S3

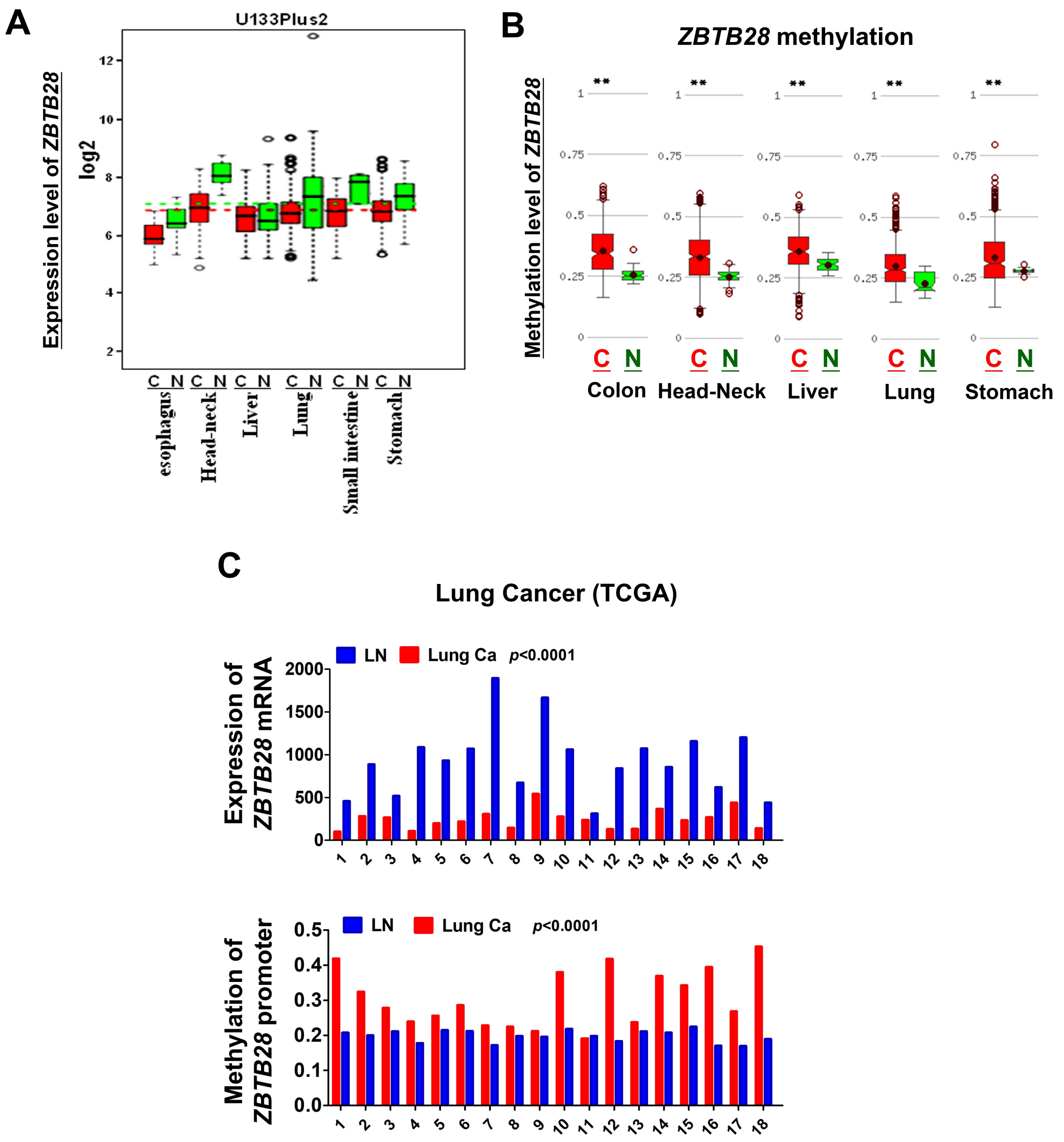
A
U133Plus2
Expression level of ZBTB28
log2
C N C N C N C N C N C N
esophagus
Head-neck
Liver
Lung
Small intestine
Stomach
B
ZBTB28 methylation
Methylation level of ZBTB28
C N
Colon
Head-Neck
Liver
Lung
Stomach
C
Lung Cancer (TCGA)
LN
Lung Ca
p<0.0001
Expression of ZBTB28 mRNA
Methylation of ZBTB28 promoter

Figure S4

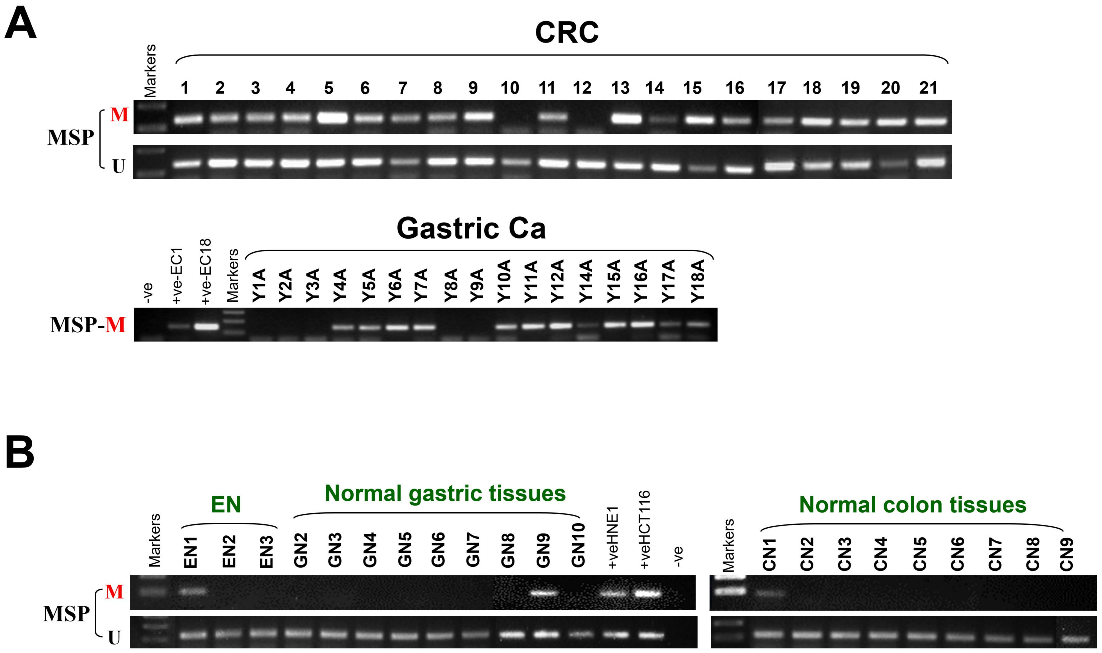
A
CRC
Markers
1 2 3 4 5 6 7 8 9 10 11 12 13 14 15 16 17 18 19 20 21
MSP
M
U
Gastric Ca
-ve
+ve-EC1
+ve-EC18
Markers
Y1A Y2A Y3A Y4A Y5A Y6A Y7A Y8A Y9A Y10A Y11A Y12A Y14A Y15A Y16A Y17A Y18A
MSP-M
B
EN
Normal gastric tissues
Normal colon tissues
Markers
EN1 EN2 EN3 GN2 GN3 GN4 GN5 GN6 GN7 GN8 GN9 GN10
+veHNE1
+veHCT116
-ve
Markers
CN1 CN2 CN3 CN4 CN5 CN6 CN7 CN8 CN9
MSP
M
U

Figure S5

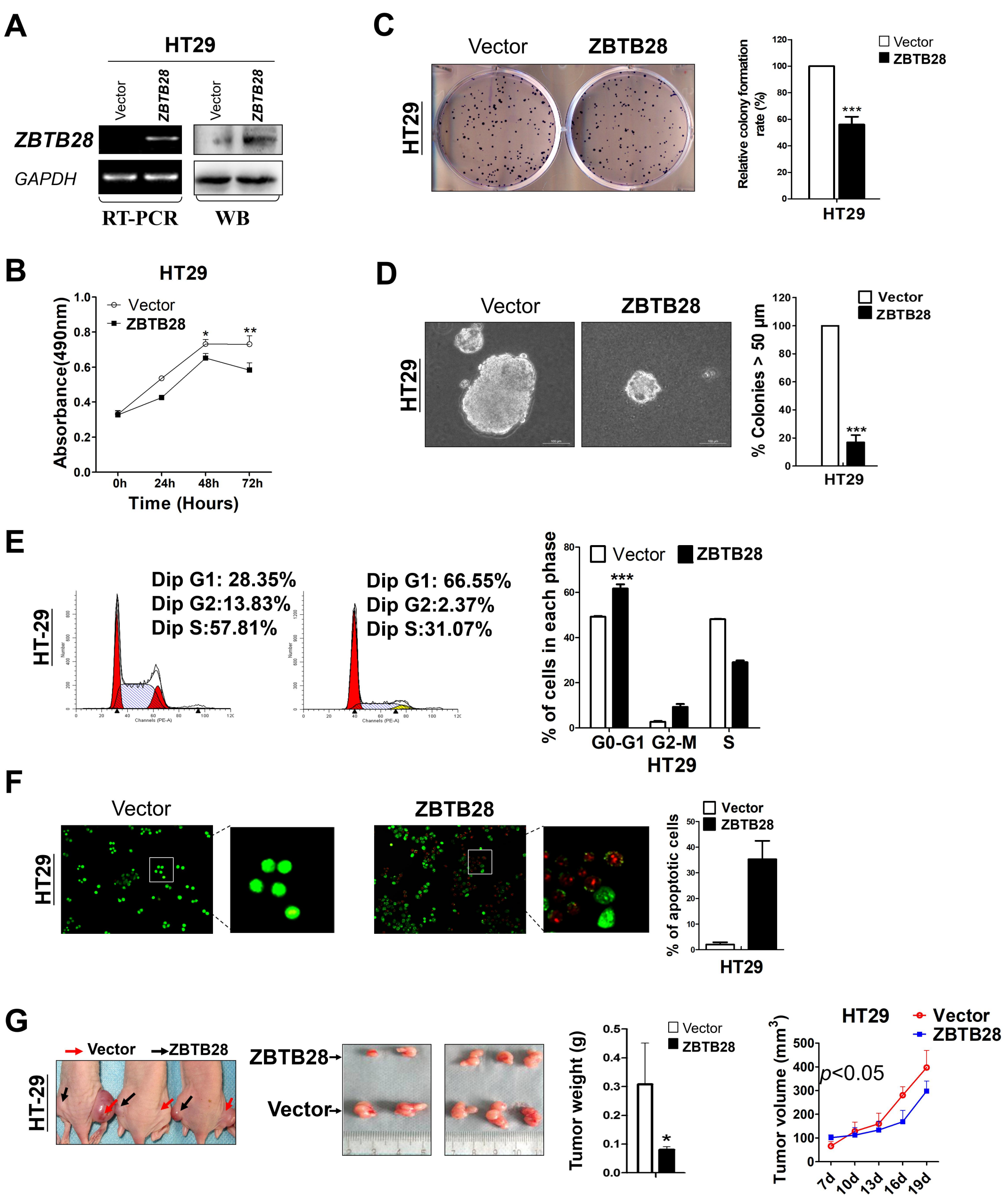
A
HT29
Vector
ZBTB28
ZBTB28
GAPDH
RT-PCR
WB
B
HT29
Vector
ZBTB28
Absorbance(490nm)
Time (Hours)
0h
24h
48h
72h
C
Vector
ZBTB28
HT29
Relative colony formation rate (%)
D
Vector
ZBTB28
HT29
% Colonies > 50 μm
E
HT-29
Dip G1: 28.35%
Dip G2:13.83%
Dip S:57.81%
Dip G1: 66.55%
Dip G2:2.37%
Dip S:31.07%
% of cells in each phase
Vector
ZBTB28
G0-G1
G2-M
S
HT29
F
Vector
ZBTB28
HT29
% of apoptotic cells
G
HT-29
Vector
ZBTB28
Tumor weight (g)
Tumor volume (mm3)
p<0.05
7d
10d
13d
16d
19d

Figure S6

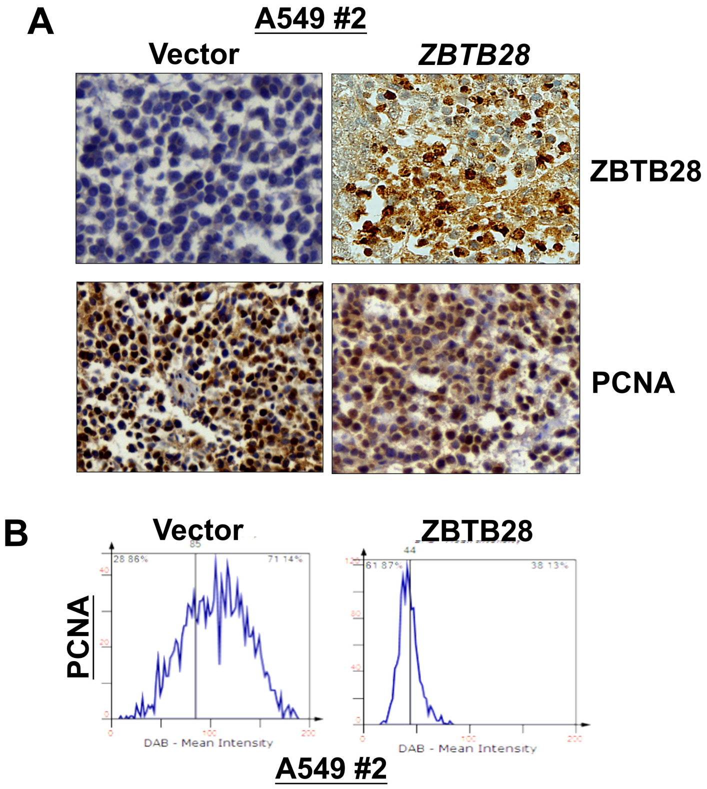
A
A549 #2
Vector
ZBTB28
ZBTB28
PCNA
B
Vector
ZBTB28
PCNA
DAB - Mean Intensity
DAB - Mean Intensity
A549 #2

Figure S7

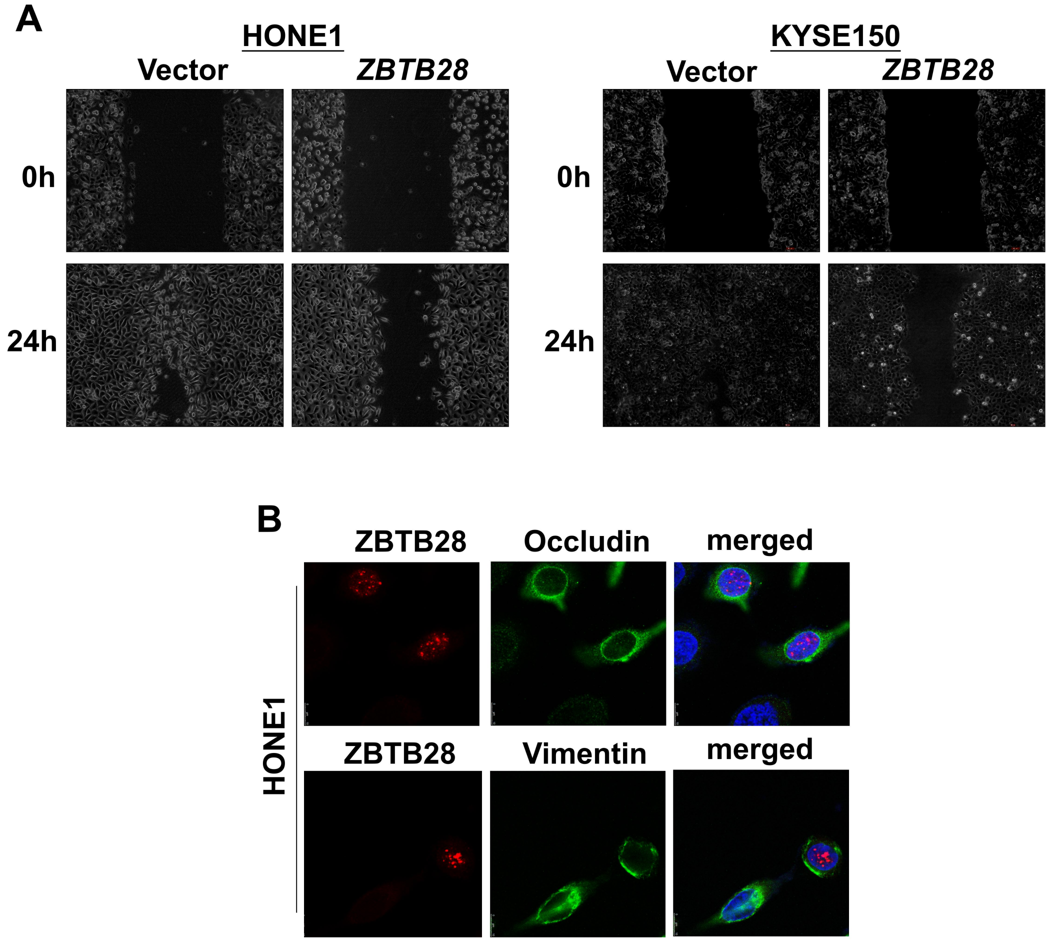
A
HONE1
Vector
ZBTB28
KYSE150
Vector
ZBTB28
0h
24h
0h
24h
B
ZBTB28
Occludin
merged
ZBTB28
Vimentin
merged
HONE1

Figure S8

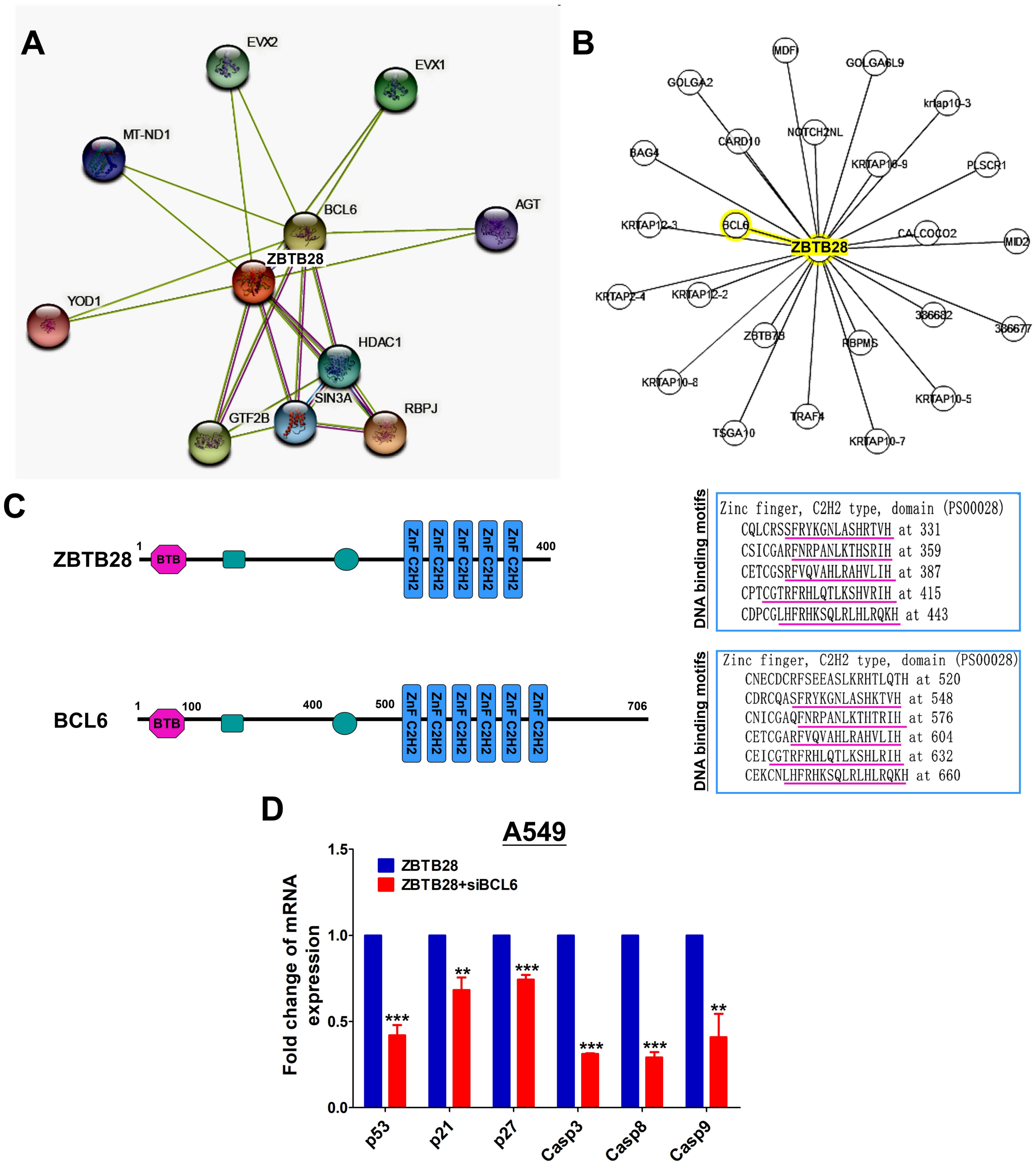
A
EVX2
EVX1
MT-ND1
BCL6
AGT
ZBTB28
YOD1
HDAC1
SIN3A
GTF2B
RBPJ
B
MDFI
GOLGA6L9
GOLGA2
krtap10-3
NOTCH2NL
CARD10
BAG4
KRTAP10-9
PLSCR1
KRTAP12-3
BCL6
ZBTB28
CALCOCO2
MID2
KRTAP2-4
KRTAP12-2
386682
386677
ZBTB8
RBPMS
KRTAP10-8
KRTAP10-5
TRAF4
TSGA10
KRTAP10-7
C
ZBTB28
BTB
ZnF C2H2
400
BCL6
1
100
400
500
706
DNA binding motifs
Zinc finger, C2H2 type, domain (PS00028)
CQLCRSSFRYKGNLASHRTVH at 331
CSICGARFNRPANLKTHSRIH at 359
CETCGSRFVQVAHLRAHVLIH at 387
CPTCGTRFRHLQTLKSHVRIH at 415
CDPCGLHFRHKSQLRLHLRQKH at 443
DNA binding motifs
Zinc finger, C2H2 type, domain (PS00028)
CNECDCRFSEEASLKRHTLQTH at 520
CDRCQASFRYKGNLASHKTVH at 548
CNICGAQFNRPANLKTHTRIH at 576
CETCGARFVQVAHLRAHVLIH at 604
CEICGTRFRHLQTLKSHLRIH at 632
CEKCNLHFRHKSQLRLHLRQKH at 660
D
A549
ZBTB28
ZBTB28+siBCL6
Fold change of mRNA expression
1.5
1.0
0.5
0.0
p53
p21
p27
Casp3
Casp8
Casp9

Figure S9

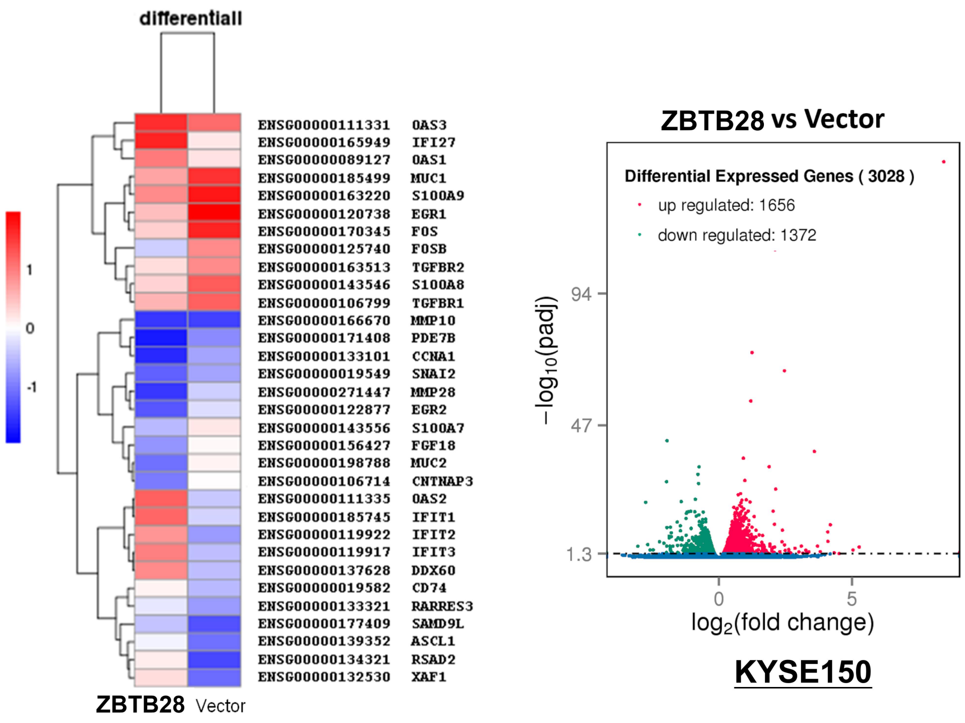
differentiall
1
0
-1
ENSG00000111331 OAS3
ENSG00000165949 IFI27
ENSG00000089127 OAS1
ENSG00000185499 MUC1
ENSG00000163220 S100A9
ENSG00000120738 EGR1
ENSG00000170345 FOS
ENSG00000125740 FOSB
ENSG00000163513 TGFBR2
ENSG00000143546 S100A8
ENSG00000106799 TGFBR1
ENSG00000166670 MMP10
ENSG00000171408 PDE7B
ENSG00000133101 CCNA1
ENSG00000019549 SNAI2
ENSG00000271447 MMP28
ENSG00000122877 EGR2
ENSG00000143556 S100A7
ENSG00000156427 FGF18
ENSG00000198788 MUC2
ENSG00000106714 CNTNAP3
ENSG00000111335 OAS2
ENSG00000185745 IFIT1
ENSG00000119922 IFIT2
ENSG00000119917 IFIT3
ENSG00000137628 DDX60
ENSG00000019582 CD74
ENSG00000133321 RARRES3
ENSG00000177409 SAMD9L
ENSG00000139352 ASCL1
ENSG00000134321 RSAD2
ENSG00000132530 XAF1
ZBTB28 Vector
ZBTB28 vs Vector
Differential Expressed Genes (3028)
up regulated: 1656
down regulated: 1372
94
47
1.3
$-\log_{10}(\text{padj})$
0
5
$\log_2(\text{fold change})$
KYSE150
